# Functionality Enhancement of Pullulan‐Based Composites for Food Packaging Applications

**DOI:** 10.1111/1541-4337.70349

**Published:** 2025-12-08

**Authors:** Bibek Bahadur Shrestha, Jayeeta Mitra, Saji George

**Affiliations:** ^1^ Agricultural and Food Engineering Department Indian Institute of Technology Kharagpur Kharagpur India; ^2^ Department of Food Science and Agricultural Chemistry McGill University, Macdonald‐Stewart Building, Macdonald Campus Québec Canada

**Keywords:** bioactive compounds, chemical modifications, electrospinning, encapsulation, pullulan, smart packaging

## Abstract

Contemporary research in food packaging is focused on developing sustainable alternatives to petroleum‐based materials. Pullulan, a microbial biopolymer traditionally employed as a food additive, is harnessing interest for food packaging applications due to its exceptional film‐forming ability, biodegradability, and nontoxic nature. However, there are key limitations associated with the cost of production and suboptimal physicochemical attributes (e.g., inadequate water barrier and mechanical strength) that curtail the successful industrial translation of pullulan as a packaging polymer. Accordingly, this review examines effective ways for boosting biosynthetic efficiency of pullulan production through genetic and metabolic engineering of native strains and identifies emerging strategies such as targeted chemical modifications, electrospinning, incorporation of bioactive compounds, and film casting to enhance properties of pullulan‐based packaging materials. Encapsulation strategies for bioactive substances are emphasized in pullulan‐based active packaging for controlled release and sustained efficacy, whereas integration with pH‐responsive sensing entities enables smart packaging for real‐time freshness monitoring of protein‐rich foods. Further, we examined regulatory and safety frameworks, providing a perspective that bridges innovation with compliance requirements for commercial deployment. All in all, this review demonstrates the potential to reduce production costs and improve film properties, which has significantly strengthened the prospects of pullulan as a sustainable, biopolymer‐based alternative to synthetic materials.

## 1 Introduction

Packaging is a critical unit operation across the supply chain, playing a vital role in ensuring food safety by shielding products from chemical and microbiological contamination, physical damage, and quality degradation (Yashwanth et al. 2025). Food shelf life is typically reduced by factors such as moisture, oxygen, light exposure, and microbial spoilage, highlighting the need for innovative and effective packaging solutions (Nilsen‐Nygaard et al. 2021; Corato 2020). Driven by the increasing demand for packaged foods, the global packaging industry saw a rise in revenue, growing from $42.5 billion in 2014 to approximately $48.3 billion by 2020 (Cruz et al. 2022). Currently, the majority of the food packaging demand is being fulfilled by fossil fuel‐derived plastic packaging materials (George et al. 2020). Evidently, plastic packaging materials lead to environmental sustainability issues through the sheer amount of waste generated and discarded, which has a substantial impact on global warming, carbon footprint, and ecosystems (Ghosh and Jones 2021; Rajendran et al. 2025).

The market for packaging materials derived from sustainable and eco‐friendly sources has seen significant growth, driven by the priority to minimize environmental impact (Taherimehr et al. 2021). Biopolymers are intensively being explored as alternatives for food packaging materials owing to their degradability, biocompatibility, and overall environmental sustainability (Matheus et al. 2023; Moeini et al. 2021). However, packaging materials developed from single biopolymers often exhibit inferior mechanical, barrier, and thermal properties compared to synthetic polymers (Bhargava et al. 2020), limiting their use for commercial food packaging applications.

Pullulan has been pervasively utilized by the food industry for various applications, such as texturizing, stabilizing, gelling, and thickening agents, for many years (Ghosh et al. 2022). In addition to these applications, pullulan is extensively utilized in biomedical engineering, making it suitable for the development of drug delivery systems with controlled and targeted release (Le et al. 2022), scaffolds for tissue engineering that facilitate cell adhesion and proliferation (Qin et al. 2021), and wound healing materials that enhance tissue regeneration while inhibiting microbial infection (Chen et al. 2022). Furthermore, pullulan has been incorporated into skin‐whitening formulations for managing various pigmentation disorders, including sunburn‐induced hyperpigmentation, freckles, chloasma, and Addison's disease (Coltelli et al. 2020). Pullulan contributes to cholesterol reduction and exerts prebiotic effects by selectively stimulating the growth of beneficial intestinal microbiota, functioning as a soluble dietary fiber (Savitskaya et al. 2024). Furthermore, its low digestibility renders it suitable for the formulation of low‐calorie and diabetic‐friendly foods, thereby expanding its applicability in the development of functional and health‐promoting food products (Muthusamy et al. 2022). These studies highlight the health benefits and nontoxic nature of pullulan, which has been Generally Recognized as Safe (GRAS) by the US Food and Drug Administration (FDA) for use in foods (Goswami et al. 2021).

Production of pullulan by black yeast, *Aureobasidium pullulans* (formerly referred to as *Pullularia pullulans*), was initially identified by Bauer in 1938, with its characteristics and isolation process detailed couple of years later by Bernier (Aquinas et al. 2024). *A. pullulans* is a nonpathogenic microorganism found in animal tissues, forest soils, freshwater, and seawater. The initial industrial pullulan production was undertaken by Hayashibara Company Limited, located in Okayama, Japan, in 1976, and it was introduced to the market in 1982 as binder for processed marine products and thickener for dressings and sauces or as edible film (Das and Chakraborty 2020). The global pullulan market is witnessing steady growth, with its valuation expected to rise from around $68 million in 2022 to approximately $89 million by 2028, reflecting a projected compound annual growth rate of 4.5% over the period (Cruz‐Santos et al. 2023). The Asia‐Pacific holds the largest share of the market, accounting for over 50%, whereas North America accounts for approximately 30% (Absolute Reports 2024). The pharmaceutical sector constitutes the predominant segment of the pullulan market, succeeded by the food and cosmetic industries (Aquinas et al. 2024).

Given its remarkable film‐forming capabilities and amenability to modification, pullulan is now being explored as a biopolymer for fabricating food packaging materials (Ghosh et al. 2022). Nevertheless, pullulan‐based films are subject to some constraints, notably brittleness, hydrophilicity, lack of active functions such as antibacterial or antifungal activity, and comparatively higher cost of production (Trinetta and Cutter 2025). Recent studies addressing these key limitations of pullulan are being reported in literature. Evidently, the physicochemical characteristics of pullulan, including thermal stability, mechanical, optical barrier, and vapor or oxygen barrier (OB) properties, can be enhanced through chemical modification of pullulan, such as octenylsuccination (Omar‐Aziz et al. 2021), as well as the incorporation of plasticizers or fillers (Zamanidehyaghoubi et al. 2025). Several pullulan‐based composites have been studied for improving film properties. For instance, pullulan was blended with cassava starch (Sapper et al. 2019) or carboxymethyl cellulose (Thangavelu and Kulandhaivelu 2022) to enhance the elasticity of the film. The incorporation of bacterial cellulose (Ding et al. 2022) or cellulose nanofibers (Riahi et al. 2024) into pullulan was found to reinforce film strength. Similarly, blending pullulan carboxylated cellulose nanocrystals (Chen and Chi 2021) or lignin (Ding et al. 2024) improved the water barrier properties of films. On the other hand, to impart antibacterial activity, pullulan was blended with chitosan (Gan et al. 2023). Undoubtedly, the incorporation of bioactive substances, namely, thymol (Zhang et al. 2025), carvacrol (Ertan et al. 2023), curcumin (Khan et al. 2022), allicin (Jia et al. 2022), and zanthoxylum bungeanum essential oil (Qin et al. 2022), was reported to improve the functional properties of pullulan‐based films. Studies have also shown that the addition of active substances can alter the physicochemical properties. For example, gallic acid (Gasti et al. 2023) enhances water resistance, whereas citric acid (Sharmin et al. 2022) improves the elasticity of the film. Apart from the shortcomings of native pullulan fiber, its widespread applicability has been curtailed by cost of production. Primary factors contributing to the relatively higher costs of pullulan manufacturing include the cost of fermentation media components, namely, glucose, sugar alcohol, glycerol, and organic acid, along with the use of organic solvents, namely, ethanol and methanol, for the recovery of pullulan from the fermentation broth (Cruz‐Santos et al. 2023). Agro‐based wastes are viable alternatives with the potential to substantially reduce production costs and are also environmentally sustainable.

Although literature reviews have underscored the potential of pullulan in sustainable food packaging (Ghosh et al. 2022; Dewan and Islam 2024; Rashid et al. 2024; Chaari and Smaoui 2024; Bhari et al. 2025), the high cost of large‐scale production has hindered its widespread commercial application. Furthermore, existing studies have primarily focused on pullulan‐based edible coatings and composite active packaging, without addressing critical aspects such as mitigating the inherent water sensitivity, imparting functional characteristics into native pullulan film, and controlling the release of active substances from pullulan‐based films. To bridge these gaps, this review emphasizes strategies for genetically modifying fungal strains to enhance pullulan yield and reduce production costs through the utilization of agrowaste. In parallel, this review examines chemical modification approaches, such as esterification, etherification, oxidation, and amidification, for tailoring the physicochemical and functional attributes of pullulan to meet the demands of modern food packaging applications. Recent advancements in pullulan‐based edible coatings and packaging films are critically analyzed, with particular attention to encapsulation techniques that ensure controlled release and long‐term stability of bioactive compounds in packaging systems. This review uniquely discusses key challenges, future research directions, and regulatory frameworks. Overall, this review adopts a comprehensive perspective, integrating production, functionalization, application, and regulatory considerations to guide stakeholders with actionable insights for future research and the commercial translation of pullulan‐based sustainable food packaging materials.

## 2 Chemical Structure of Pullulan

**FIGURE 1:**
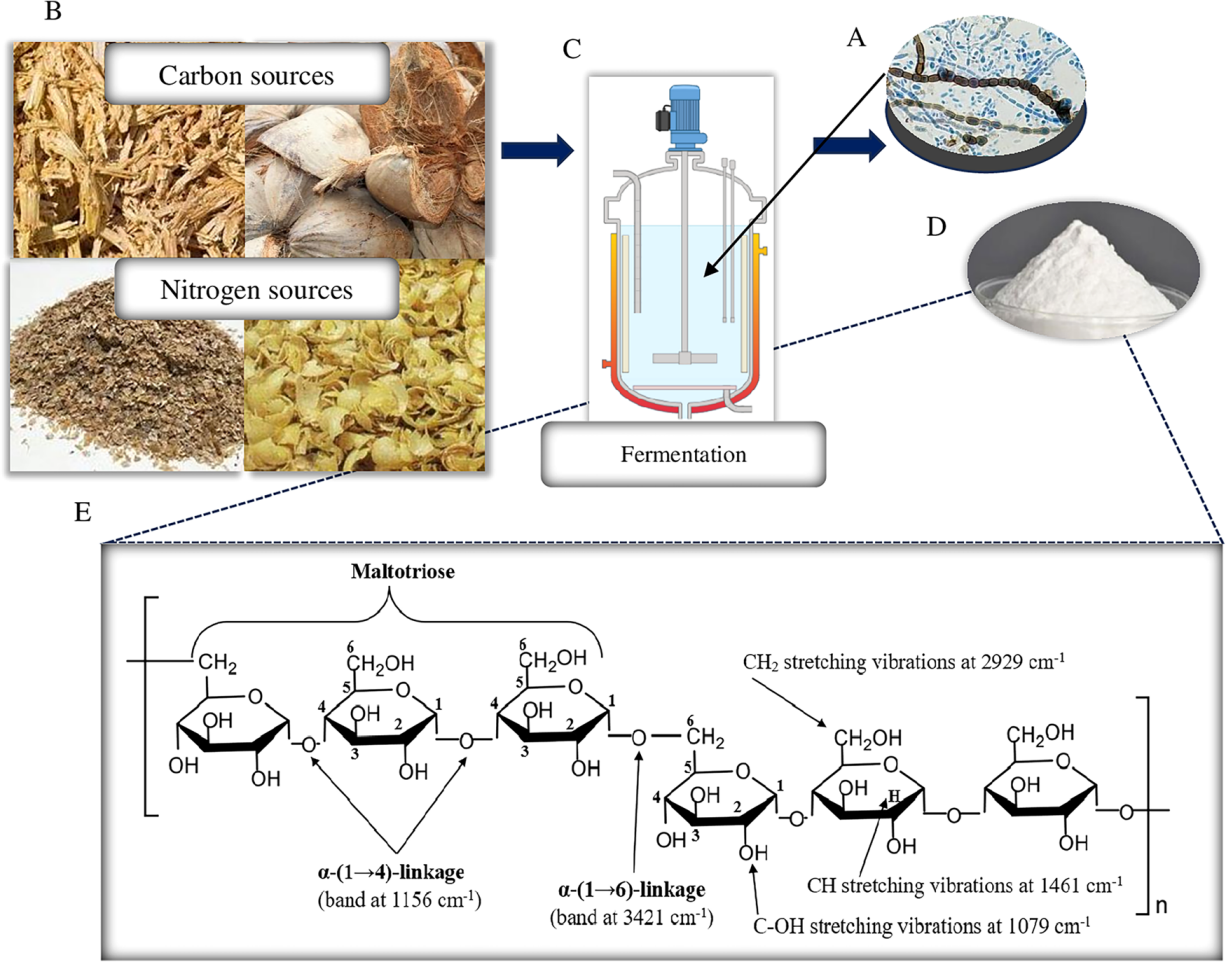
Pullulan is a biopolymer (A) obtained from microorganisms such as *Aureobasidium pullulans*, which are being genetically modified or adapted for improving the yield of pullulan during fermentation; (B) secondary raw materials from agri‐sector (coconut husk, sugarcane bagasse) are used to substitute fermentation media to reduce the cost of production; (C) pullulan polymers secreted into fermentation media; (D) later processed to obtain pullulan as white powder; (E) chemically pullulan is a repetitive maltotriose unit connected to each other by α‐(1→6)‐linkages, and glucose molecules are connected by α‐(1→4)‐linkages, establishing one maltotriose unit.

Chemically, pullulan is a polymer of maltotriose units connected to each other by α‐(1→6)‐glycosidic bonds with the proposed chemical formula (C_6_H_10_O_5_)*_n_* (Ghosh et al. 2022). Nonionic pullulan is made up of linear and unbranched maltotriose repeating units. Its chemical formula is [α‐d‐glucopyranosyl‐(1→4)‐α‐d‐glucopyranosyl‐(1→4)‐α‐d‐glucopyranosyl‐(1→6)]*_n_* (Mulla et al. 2023). The α‐(1→4)‐linkages integrate three glucose moieties and establish one maltotriose unit, whereas α‐(1→6)‐linkage binds maltotriose, forming a repeated maltotriose unit. The α‐(1→6)‐linkages in pullulan are hydrolyzed by the pullulanase enzyme, yielding the oligomers. Analysis of pullulan structure is often carried out using Fourier‐transform infrared (FTIR), nuclear magnetic resonance (NMR) spectroscopy, and chromatographic techniques. Peaks at 1156 and 3421 cm^−1^ from FTIR confirm the existence of α‐(1→4)‐ and α‐(1→6)‐linkages, respectively, whereas that at 1079 cm^−1^ indicates the existence of –OH stretching vibrations in pullulan structure (Singh and Kaur 2019). NMR spectroscopy can be used for quantifying the proportion of α‐(1→6)‐ and α‐(1→4)‐linkages in pullulan. The ^1^H NMR spectrum of pullulan has two distinct resonances associated with the anomeric protons at around *δ* 5 and *δ* 5.5 ppm, corresponding to α‐(1→6)‐ and α‐(1→4)‐linkages, respectively (Hernandez‐Tenorio and Giraldo‐Estrada 2022). Figure 1 presents the raw material used for deriving fermentation media and the general chemical structure of pullulan obtained through fermentation.

## 3 Properties of Pullulan

Pullulan is often regarded as an intermediary between dextran and amylose, owing to the presence of alternating α‐(1→6)‐ and α‐(1→4)‐linkages within a single component (Rashid et al. 2024) (Figure 1). The hydrophilic nature (water contact angle ∼32.1° < 90°) (Omar‐Aziz et al. 2021) and higher water absorption capacity of pullulan are mostly attributed to the presence of –OH groups, which facilitate stronger hydrogen bonds (Rai et al. 2021). In addition to its notable propensity for water solubility, OH groups facilitate the distinctive intermolecular linking pattern of pullulan, responsible for its exceptional structural elasticity when plasticized into film. The α‐(1→6)‐linkages, especially the C_1_‐O‐CH_2_(C_6_)‐linkage, exhibit greater elasticity in contrast to the C_1_‐O‐C_4_‐linkage, wherein C_1_ and C_4_ bond with glucopyranose rings (Souza et al. 2023). Eventually, this phenomenon gives rise to the formation of a disorganized and nonuniform configuration of the pullulan framework inside solution, leading to the manifestation of a grouped configuration of molecules. Plasticized native pullulan film exhibits an x‐ray diffraction pattern typical of amorphous polymer with a wide diffraction peak centered at 2*θ* = 18.4° (Haghighatpanah et al. 2020). Thus, the exceptional flexibility and the lack of crystallinity of pullulan make it suitable for the production of standalone films, flexible coatings, nanomaterials, and electrospun nanofibers. The mechanical characteristics, such as tensile strength (TS) and elongation at break (EB) of pullulan‐based film, are affected by its molecular weight (MW) and the inclusion of plasticizers or fillers. Commercially made pullulan exhibits a relatively low MW (4.2 × 10^5^), whereas the one derived from *A. pullulans* typically exhibits a higher MW (32.3 × 10^5^) (An et al. 2019). TS of low and high MW of native pullulan are 49.43 and 55.47 MPa, whereas EB values correspond to 3.09% and 4.67%, respectively (Chen et al. 2017).

Native pullulan possesses high water vapor permeability (WVP) (5.94 × 10^−11^ g/(cm s Pa)), mostly attributed to its hydrophilic characteristics, as the hydroxyl (–OH) groups in pullulan establish hydrogen bonds with H_2_O molecules (Chen et al. 2017). Alongside, pure pullulan film exhibits an oxygen permeability (OP) value of approximately 5.56 × 10^−17^ mL cm/(cm^2^ s Pa) (Xiao et al. 2015). Native pullulan film showed transmittance values of 91% and 73% at wavelengths of 660 and 280 nm, respectively (Priyadarshi et al. 2022). UV transmission within this specific range has the potential to impact lipid oxidation in food, influencing its shelf life. Native pullulan powder appears to be white in color because of its high L* value of ∼95.72.

Pure pullulan films exhibit thermoplastic behavior, softening upon heating and allowing heat sealability at temperatures around 120–130°C (Zhao et al. 2024). The thermal degradation trend of the plasticized pullulan film exhibits a distinct weight reduction, characterized by glass transition, initial, and peak breakdown temperatures of 160°C, 250°C, and 300°C, respectively, followed by a residue at 400°C, which accounts for approximately 14% of the initial mass (Vuddanda et al. 2017). This degradation pattern indicates the removal of water, disruption of glycosidic bonds, and charring, successively.

## 4 Pullulan Sources and Processing

Pullulan is generally produced by a polymorphic yeast‐like microfungus, *A. pullulans*, naturally found in temperate and tropical climates, grown using hydrolyzed starch as the substrate (Wani et al. 2021). *A. pullulans* exhibits several morphological characteristics, including immature blastospores, yeast‐like cells, chlamydospores, enlarged blastospores, and mycelia (Rensink et al. 2024). This species of yeast utilizes three different enzymes: glucosyltransferase, uridine diphosphate glucose pyrophosphorylase (UDPG‐pyrophosphorylase), and α‐phosphoglucomutase during pullulan production (Wani et al. 2021). Glucose 6‐phosphate and fructose‐6‐phosphate are converted into glucose‐1‐phosphate by the action of hexokinase and isomerase enzymes. This is followed by the enzymatic conversion of glucose‐1‐phosphate into pullulan precursor uridine diphosphate (UDP) glucose through the action of UDPG‐pyrophosphorylase enzyme. The subsequent stage entails the production of lipid‐linked matrices by combining d‐glucose residues. Ultimately, the residues undergo a reaction with isomaltosyl, resulting in pyranosyl or isopanosyl residues that contribute to the polymerization of pullulan chains (He et al. 2021). The transformation of glucose‐6‐phosphate into glucose‐1‐phosphate is facilitated by the α‐phosphoglucomutase enzyme. Besides *A. pullulans*, other microorganisms are also utilized in the production of pullulan, including *Cryphonectria parasitica*, *Tremella mesenterica*, *Rhodotorula bacarum*, and *Teloschistes flavicans* (Singh and Kaur 2019). Improving the yield of pullulan during fermentation is a crucial determinant for the commercial‐scale application.

## 5 Strategies to Enhance Pullulan Production Yield

### 5.1 Genetic Manipulation

Natural species often exhibit limited pullulan productivity under industrial conditions due to inefficient substrate utilization, low sugar tolerance, diversion of metabolic flux toward undesired byproducts such as melanin, and weak stress resilience (Wei et al. 2021). Recent advances in genetic and metabolic engineering enable improved pullulan biosynthesis by manipulating key regulatory and biosynthetic pathways. A study reported by Gao et al. (2025) compared transcriptomic analysis of *A. pullulans* and its modified strain, identifying differentially expressed genes involved in gluconeogenesis, sucrose metabolism, and amino acid pathways. Overexpression of the glycosyltransferase gene resulted in an 8.1% increase in pullulan production, whereas overexpressing the transmembrane transporter gene reduced the MW of the polymer by 25%, facilitating better secretion through improved membrane fluidity. Similarly, Hansali et al. (2024) enhanced pullulan biosynthesis in *Aureobasidium melanogenum* by overexpressing the *PUL1* gene, achieving a 51.3% increase in polymer concentration and improved molecular uniformity, demonstrating the importance of pullulan synthetase regulation. Hamidi et al. (2019) also highlighted that optimizing the expression of genes involved in UDP‐glucose biosynthesis could elevate pullulan yields by more than 40%, underlining the importance of precursor pathway control.

In parallel, classical strain improvement strategies have shown remarkable promise; for example, Zhang et al. (2024) developed a UV‐mutated *A. pullulans* that boosted pullulan production from 26.5 to 76.88 g/L by accelerating sugar metabolism and enhancing cell performance. In another approach, Li et al. (2023) combined mutagenesis with adaptive laboratory evolution to engineer a hypertonic‐tolerant strain capable of thriving in up to 200 g/L glucose, yielding 61.2 g/L of pullulan compared to 38.4 g/L from the wild‐type strain. Liu et al. (2021) further explored the metabolic interplay between pullulan and melanin production, revealing that high pullulan concentrations (≥40 g/L) induced morphological transitions, such as increased filamentous growth and melanin accumulation. Interestingly, melanin biosynthesis, though typically considered an undesired byproduct due to downstream processing challenges, was associated with a 15% to 20% enhancement in pullulan yield, suggesting a novel regulatory link between secondary metabolism and exopolysaccharide synthesis (Eldadamony et al. 2024). In addition to manipulating microbial metabolism through genetic or nutrient modifications, the cost of pullulan production could be reduced substantially (Mishra et al. 2023). In addition to genetic modification or adaptation of pullulan‐producing microorganisms for improved yield, the cost of production can be brought down by optimizing the fermentation conditions.

### 5.2 Optimization of Fermentation Conditions

Hydrolyzed starch is the primary substrate used in the manufacturing of pullulan (Haghighatpanah et al. 2021; Lin et al. 2022). However, the high cost of production has driven researchers to explore alternative substrates, especially for the nitrogen and carbon sources required for pullulan synthesis (Akdeniz Oktay et al. 2022). Agro‐industrial waste, which is abundant and often underutilized, presents a promising solution to reduce production costs and enhance sustainability in pullulan biosynthesis. Several studies have highlighted the potential of using waste materials such as sugarcane bagasse hydrolysate (Tagne et al. 2024), cassava starch (Viveka et al. 2021), grape pomace extract (Canalejo et al. 2021), hazelnut husk (Akdeniz Oktay et al. 2023), brewery waste (Oskay 2022), artichoke tubers (Xia et al. 2017), rice bran (Singh et al. 2020), and whey (Zikmanis et al. 2020) as carbon sources for pullulan production. For nitrogen sources, agro‐industrial by‐products, such as sesame seed oil cake (Mirzaee et al. 2020), corn steep liquor (Zarei et al. 2020), honey (Chen et al. 2020), and soybean pomace (Cruz‐Santos et al. 2023), have been suggested as viable alternatives. For instance, sugarcane bagasse hydrolysate as a carbon source resulted in a 20% increase in pullulan yield compared to traditional starch‐based media (Hilares et al. 2019). Similarly, Sharma et al. (2013) reported that corn steep liquor can replace synthetic nitrogen sources, leading to a 12% reduction in production costs while maintaining comparable pullulan yields.

The potential of these secondary raw materials aligns with the concept of a circular bioeconomy, where waste from the agri‐food industry is transformed into valuable products. Industries can reduce environmental impact, lower dependency on primary agricultural crops, and foster a more sustainable production model by utilizing these waste streams for pullulan production. This shift not only reduces production costs but also closes the loop in the agri‐food sector by reusing what would otherwise be discarded, advancing the goals of a circular bioeconomy. Overall, integration of these approaches could increase the yield and efficiency of pullulan production. Although the cost of pullulan as a polymer may not compare favorably with that of fossil fuel‐based synthetic polymers for producing packaging materials, it scores high in terms of biocompatibility and environmental degradation. The following section examines the suitability of native pullulan for meeting the demands of modern food packaging systems, with a focus on chemical modifications that enhance its physicochemical and functional properties.

## 6 Chemical Modifications of Pullulan

**FIGURE 2:**
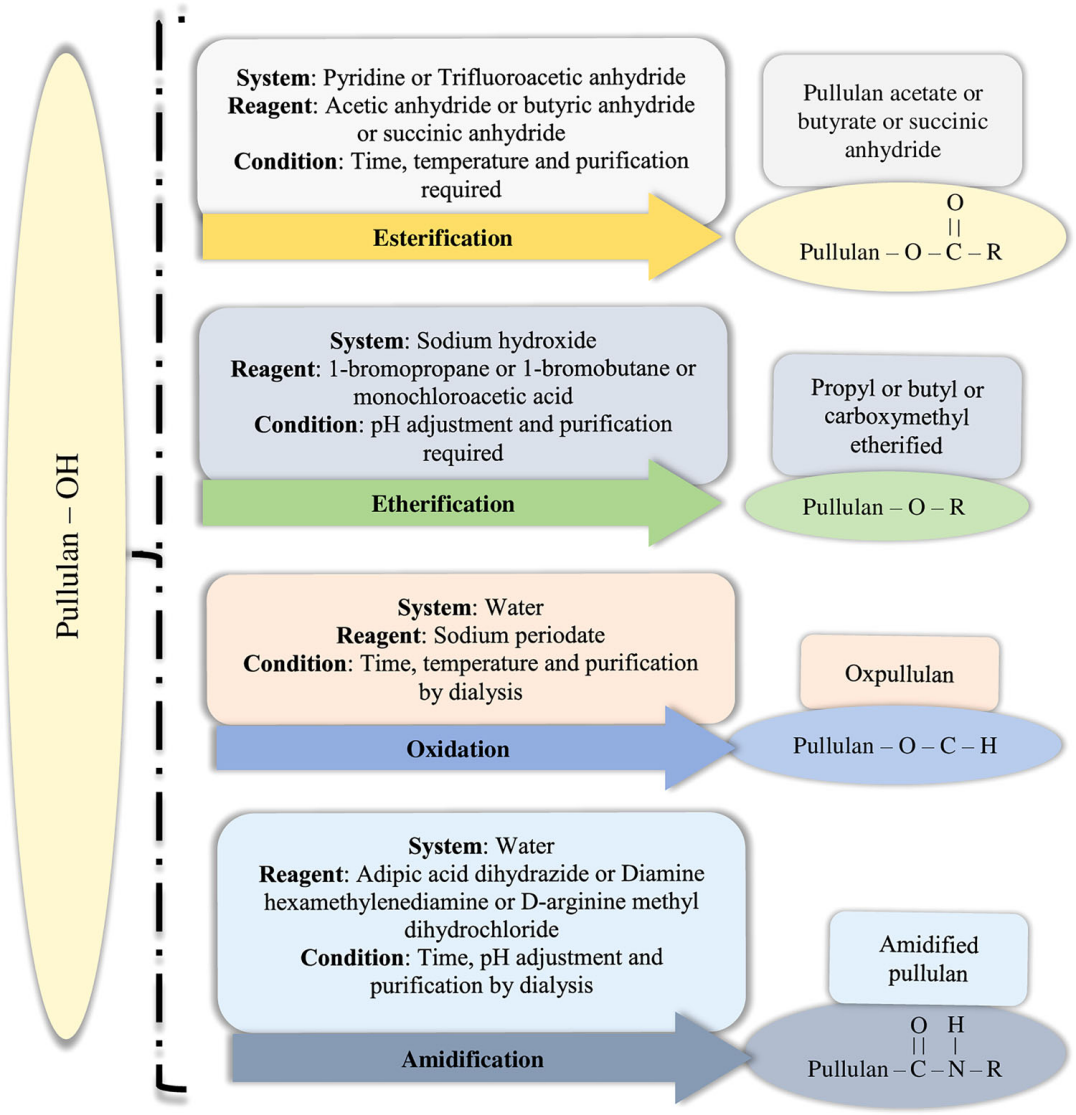
Chemical modifications on backbone of pullulan have been reported to improve its physicochemical and functional properties. This includes esterification, etherification, oxidation, and amidification.

Native pullulan fails to create a potential solution for biobased packaging film owing to its low mechanical properties, hydrophilic nature, and being neutral to microbial activities (Dewan and Islam 2024). This led to a surge in research studies addressing chemical modification of pullulan to enhance its mechanical and functional characteristics. Abundancy of hydroxyl groups (9 hydroxyl groups/monomer) facilitates chemical modifications and grafting of functional groups on the pullulan backbone structure to create desired functionalities (Tiwari et al. 2019). Hydroxyl groups are differentiated based on their spatial arrangement on the glucosidic moiety, namely, at OH‐2, OH‐3, OH‐4, and OH‐6, with ratios of 3, 3, 1, and 2 (Valachová and Šoltés 2021). These groups can undergo derivatization by esterification, etherification, oxidation, amidification, and copolymerization (Niu et al. 2019). Nevertheless, it is imperative to conduct further investigation into the research on edible coating, with modified pullulan to ensure their functional benefits are safe to the consumer. Detailed information about different pullulan derivatives is presented in Figure 2.

### 6.1 Pullulan Esterification

The esterification of pullulan is a chemical modification procedure designed to improve the physicochemical properties by incorporating ester groups (R–O–CO–R′) into its molecular framework (Ragavan et al. 2022). The process involves the reaction of the hydroxyl (–OH) groups on the glucose units of pullulan with an acid or an acid derivative (e.g., acid anhydride or acid chloride) (Omar‐Aziz et al. 2020). Primary hydroxyl groups (OH‐6) exhibit greater reactivity in esterification reactions compared to secondary hydroxyls (OH‐2 and OH‐3), as secondary hydroxyls are bonded to carbons that are linked to two additional carbons, resulting in increased steric hindrance (Kim and Jung 2022). During esterification of pullulan, the hydrogen bond network breaks down, making it water‐insoluble. Esterification is generally characterized by NMR spectroscopy to study the degree of substitution (DS). Niu et al. (2019) prepared pullulan acetate, pullulan butylate, and pullulan propionate by adding acetic anhydride, butyric anhydride, and propionic anhydride, respectively. The addition of anhydride is confirmed by reduced O–H stretching vibration situated at 3440 cm^−1^, and stronger ester group at 1739 cm^−1^, revealing higher DS of pullulan. Although all pullulan ester films displayed lower TS compared to native pullulan, pullulan propionate possessed the highest TS and EB. The water vapor barrier (WVB) of pullulan butylate was the best, followed by pullulan propionate. This is because esterified pullulan develops discontinuous particles during film formation, increasing tortuosity and resisting transmission. Similarly, pullulan butylate also showed excellent OB property, followed by pullulan propionate. Addition of *n*‐octenyl succinic anhydride (OSA) into pullulan to coat *Manilkara zapota* showed 15% weight loss compared to control (23%) after 9 days due to a 30% reduction in WVP (Shah et al. 2016). Similarly, Omar‐Aziz et al. (2021) used OSA with chickpea protein isolate and pullulan for blend film, and they reported that barrier and mechanical properties were significantly improved (*p* < 0.05).

The esterification technique has demonstrated significant improvements in barrier performance due to the introduction of hydrophobic functional groups that reduce WVP and hydrophilicity of native pullulan. The introduction of ester groups generally reduces the hydrogen bonding interactions within the polymer chain, which can compromise mechanical properties, such as TS and EB of native pullulan. Furthermore, excessive esterification can lead to structural irregularities, increased brittleness, and decreased film‐forming ability. This may happen because the substitution of –OH groups with bulky ester moieties disrupts the regular hydrogen bonding network, resulting in a stiffer matrix and brittle films. However, this method is simple and industrially feasible due to straightforward reaction mechanisms, low‐cost reagents, and ease of controlling the DS. Esterification is the most effective and secure technique for producing packaging films, particularly using GRAS reagents.

### 6.2 Pullulan Etherification

In etherification, pullulan's hydroxyl groups (–OH) are usually substituted by the alkyl groups and create ether bonds (R–O–R′) (Wang et al. 2024). Propyl‐etherified and butyl‐etherified pullulans with various DS (1.02 to 2.61) were synthesized by adding 1‐bromopropane and 1‐bromobutane, respectively (Shibata et al. 2002). FTIR spectra of the alkyl pullulans revealed that the absorption peak at 3300 cm^−1^, corresponding to stretching vibration of –OH, was weaker, confirming substitution of –OH groups in pullulan. Butyl‐etherified pullulan showed lower TS and Young's modulus than propyl‐etherified. The carbonyl carbon in the carboxyl group (–COOH) demonstrates electron shortage due to the electronegativity of the adjacent oxygen atom, signifying its electrophilic nature, allowing it to react with nucleophilic entities such as reactive oxygen species or free radicals (Deline et al. 2020). Therefore, carboxymethylated derivatives of pullulan were better at preserving blueberries (Hernandez‐Tenorio et al. 2024). Carboxymethylation results in the breakage of intermolecular and intramolecular hydrogen bonds, causing chain segments to move and facilitating the breakdown of the modified pullulan at lower temperatures. Thus, thermal stability was found to decrease with the increase in carboxymethylation in pullulan. However, OP and WVP decreased due to the change in the free volume of the polymeric chain during carboxymethylation. Coated fruits showed considerable reduction in weight loss (*p* < 0.05) due to increased hydrogen bonding between the fruit shell cuticle and coating matrix, acting as a barrier.

Overall, etherification constitutes a prospective modification technique to augment the functionality of pullulan, extending its potential as an active coating material, provided all substituents are food‐safe. Substitution with alkyl groups lowers WVP and OP of packaging material, thereby contributing to better preservation of fresh produce. The formation of denser molecular structures limits water vapor and gas transmission, and the alteration of polymer chain interactions enhances film ductility and reduces brittleness. Furthermore, etherification facilitates stronger interactions with reactive oxygen species, imparting antioxidant activity that improves food preservation. However, high degrees of substitution weaken mechanical performance and reduce thermal stability.

### 6.3 Pullulan Oxidation

The process of pullulan oxidation entails the incorporation of oxygen‐containing functional groups into the C‐6 end of pullulan (Selvakumar and Lonchin 2020). This process requires oxidizing agents, which result in the creation of aldehyde groups. In this context, Li et al. (2020a) synthesized OxPullulan by adding sodium periodate. FTIR showed the presence of aldehyde groups in OxPullulan by exhibiting novel peak at 1730 cm^−1^. The hydroxylamine hydrochloride method was employed to estimate the oxidation degree of OxPullulan, yielding 40%. Although thermal properties were reduced, OP and WVP of the film were reduced significantly (*p* < 0.05), forming more hydrogen bonds between –OH groups and water molecules. It makes them more branched polymeric chains, which provide excellent barrier properties. Similarly, Ma et al. (2025) employed sodium periodate to derive dialdehyde pullulan for the fabrication of electrospun nanofibers intended for the preservation of blueberries at room temperature. The fiber morphology revealed that the oxidation degree increased the nanofiber diameter from 71 to 170 nm, as the degree of pullulan oxidation decreased the conductivity of electrospinning solution, facilitating formation of coarser fibers under weakened electrostatic forces. Successful oxidation of pullulan was confirmed by the appearance of a new absorption peak at 1728 cm^−1^, attributed to the C═O stretching vibration of aldehyde groups. Interestingly, a higher degree of oxidation elevated the thermal degradation temperature of nanofibers, enhancing thermal stability. Furthermore, they reported that degree of oxidation increased TS but reduced EB of nanofibers, as oxidation improved network structure of nanofibers but restricted the movement of molecular chains. They reported that dialdehyde pullulan nanofibers reduced weight loss by 6%, delayed pH rise, and maintained the firmness of blueberries during storage. They further observed that nanofibers suppressed bacterial growth in blueberries, thereby extending fruit freshness and shelf life. In another study, Roy et al. (2023) synthesized oxidized pullulan using different concentrations of sodium periodate. In contrast to the characteristic FTIR peak at 1728 cm^−1^, they corroborated the presence of aldehyde groups in oxidized pullulan by ^1^H NMR spectra and ^13^C NMR spectra, which exhibited distinct signals at 9.19 and 175 ppm, respectively. They highlighted that the bactericidal activity of oxidized pullulan against *Staphylococcus aureus* was dose‐dependent, with minimum inhibitory and minimum bactericidal concentrations of 18.75 and 40.65 µg/mL, respectively.

The oxidation of pullulan introduces aldehyde functionalities, which significantly expand its potential for food packaging applications. The incorporation of carbonyl groups in pullulan enhances barrier properties by reducing OP and WVP through the formation of branched polymeric chains with stronger intermolecular hydrogen bonding. Furthermore, pullulan oxidation is associated with antibacterial properties, demonstrating its applicability in functional food packaging. Excessive oxidation in pullulan disrupts the hydrogen bonding network and accelerates the degradation at low temperature, lowering thermal stability of the oxidized film. The use of strong oxidizing agents such as sodium periodate raises concerns regarding their safety due to potential chemical migration into stored food systems, especially if residual reagents are not completely removed during purification. Therefore, biocompatibility of the film produced from oxidized pullulan should be evaluated prior to its evaluation in food packaging applications.

### 6.4 Pullulan Amidification

The positively charged amino group (–NH_2_) in the backbone of pullulan provides antibacterial activity. Cationic functional group associates with the negatively charged bacterial cell membrane electrostatically, leading to membrane rupture and intracellular component leakage, resulting in loss of viability (Gu et al. 2020). In this context, Li et al. (2020a) prepared a blend film of adipic acid dihydrazide (ADH)‐OxPullulan and chitosan by oxidizing the pullulan, followed by amidification with ADH. FTIR analysis showed the existence of a free amino group, revealing vibration of N–H stretching at 1551 and 3300 cm^−1^. The addition of ADH resulted in the disruption of the hydrogen bonding inside the pullulan, leading to a reduction in its thermal properties. However, ADH‐OxPullulan and chitosan composite presented significant functional activity against *S. aureus*. In another study, a positively charged active amino group was grafted by enamine diamine diethylenetriamine and diamine hexamethylenediamine to increase the WVB property and antibacterial characteristics (Li et al. 2020b). In addition to imparting antibacterial activity, the degree of pullulan amidification plays a crucial role in determining its biocompatibility, as excessive amidification may pose cytotoxic risks to human cells. In this context, Zhang et al. (2023) developed amidated pullulan by activating succinic anhydride‐modified pullulan with d‐arginine methyl dihydrochloride, resulting in strong antibacterial efficacy against *S. aureus* and *Escherichia coli*, with minimum inhibitory concentrations of 50 and 55 mg/mL, respectively. Furthermore, the modified film was tested on human epithelial cells to assess its cytotoxic potential. They revealed a high cell survival rate exceeding 90% at a concentration of 100 µg/mL. However, as the concentration increased to 400 µg/mL, the survival rate declined to approximately 75%. These results were attributed to electrostatic interactions between the positively charged amino groups on amidated pullulan and the negatively charged proteins and glycans on the cell surface, potentially disrupting cellular integrity and viability. These findings underscore the importance of optimizing amidification levels to balance functional performance of film with cytocompatibility.

Amidification introduces positively charged amino groups into pullulan, imparting strong antibacterial activity in packaging films through strong electrostatic interactions between the cationic polymer and bacterial cells. The functional groups in amidified pullulan films improve water barrier performance; however, they adversely affect integrity and thermal stability of film. Most importantly, the degree of amidification directly influences cell cytotoxicity in a dose‐dependent manner. Excessive introduction of amino groups can cause unfavorable interaction with human cell membranes, reducing survival rates and raising safety concerns. Therefore, amidified pullulan presents a promising route to develop functional packaging films, but its practical application demands precise optimization of substitution levels to achieve a balance between antimicrobial efficacy and biocompatibility.

### 6.5 Pullulan Copolymerization

Pullulan copolymerization denotes the chemical amalgamation of pullulan with one or more distinct monomers or polymers to produce a novel polymer material exhibiting enhanced mechanical strength, hydrophobicity, and thermal stability (Das et al. 2023). This involves initiators and catalysts to establish bonds between pullulan and the co‐monomer or polymer via free radical polymerization and atom transfer radical polymerization techniques. A greater quantity of pullulan offers a higher count of possible activation or polymerization sites; nevertheless, the initiating species constrains the development of active sites (Mert et al. 2020). Therefore, a reduced initiator/pullulan ratio may be a limiting factor for achieving higher grafting yields. There are many cases when methyl acrylate, poly(ethylene glycol), poly(methyl methacrylate), *N*‐isopropylacrylamide, 3‐acrylamidopropyl trimethylammonium chloride, and polyacrylamide are grafted onto pullulan through redox‐initiated polymerization or free radicals (Babutan et al. 2021). The application of free radical polymerization to graft poly(*N*‐vinylimidazole) (PNVI) onto pullulan resulted in enhanced thermal stability, associated with the generation of more hydrogen bonds (Hezarkhani and Yilmaz 2019). FTIR spectra confirmed copolymerization by peaks at 2922 and 3143 cm^−1^ owing to stretching vibrations of C–H and N–H groups, respectively. Additionally, thermogravimetric analysis asserted that the 20% rapid breakdown of pullulan at 325°C was inhibited following this grafting. Furthermore, pullulan‐graft‐PNVI maintained 15% of its weight at 600°C, whereas pullulan underwent complete decomposition, demonstrating the thermal stability of this copolymer. The synthesized graft copolymer formed complexes in an aqueous acidic medium, which imparted a cationic characteristic and may introduce functional characteristics of pullulan.

Copolymerization markedly enhances the intrinsic properties of native pullulan films, imparting superior mechanical strength, thermal stability, and barrier performance. The reliance on synthetic co‐monomers and radical initiators may compromise the inherent biodegradability and food safety of pullulan, challenging its positioning as a sustainable food packaging material. Moreover, the efficiency of grafting is highly sensitive to initiator ratios and reaction conditions, making process reproducibility and scalability nontrivial. A key insight is that copolymerization tends to shift pullulan away from its green identity toward a performance‐driven material, making it best suited for applications where extreme functionalities, such as high‐temperature resistance or tailored surface charge, are indispensable.

As suggested by reported studies cited in this section, surface modification of pullulan is a strategic route to enhance its physicochemical and functional limitations, including hydrophilicity, weak mechanical properties, and lack of bioactivity. However, any chemical modification aimed at edible films or coatings must undergo migration testing in accordance with regulatory food safety standards. Table 1 presents a comparative overview of these chemical modification techniques applied to pullulan. In conclusion, the choice of chemical modification method should be dictated not only by the functional enhancement desired but also by the intended application (edible vs. non‐edible), scale‐up simplicity, and most critically, the safety profile of the final product. Integrating health risk assessment, migration studies, and compliance with food contact material regulations is essential to ensure the commercial viability and consumer safety of modified pullulan‐based films.

**TABLE 1:** Comparison of different modification methods of pullulan for packaging applications.

Modification methods	Groups attached	Advantages	Disadvantages	Industrial feasibility	Best suited applications
Esterification	Acetate, butylate, propionate, succinate	Excellent water and oxygen barrier properties	Low tensile strength and elasticity compared to native pullulan film	Feasible for large‐scale production	Packaging films
		Water insolubility after modification		Cost of reagents (anhydrides) could be limiting	
		Enhanced thermal stability			
Etherification	Propyl, butyl, carboxymethyl	Improved hydrophilicity	Reduced thermal stability	Potentially feasible	Edible coatings
		Tunable mechanical properties		Needs optimization for large‐scale applications	
		Excellent gas and water barrier properties			
Oxidation	Aldehydes (via sodium periodate)	Improved barrier properties	Reduced thermal properties	Feasible with appropriate oxidizing agents	Edible coatings
				Oxidation degree and cost may limit industrial use	
Amidification	Amino groups (via adipic acid dihydrazide, diamine diethylenetriamine, diamine hexamethylenediamine)	Strong antibacterial activity	Multistep synthesis	Feasible in small‐to‐medium‐scale production	Active food packaging
		Good film‐forming capability with blends	Reduced thermal stability	Scalability may require careful control of reaction conditions	
Copolymerization	Methyl acrylate, poly(ethylene glycol), poly(methyl methacrylate)	Enhanced mechanical strength	Requires specific initiators and catalysts	Feasible with modern polymerization techniques	High performance films and thermal stable coatings
		Improved hydrophobicity and better thermal properties	Complexity in process	But cost of monomers may hinder large‐scale use	

## 7 Physical Techniques for Preparation of Pullulan‐Based Biocomposites

**FIGURE 3:**
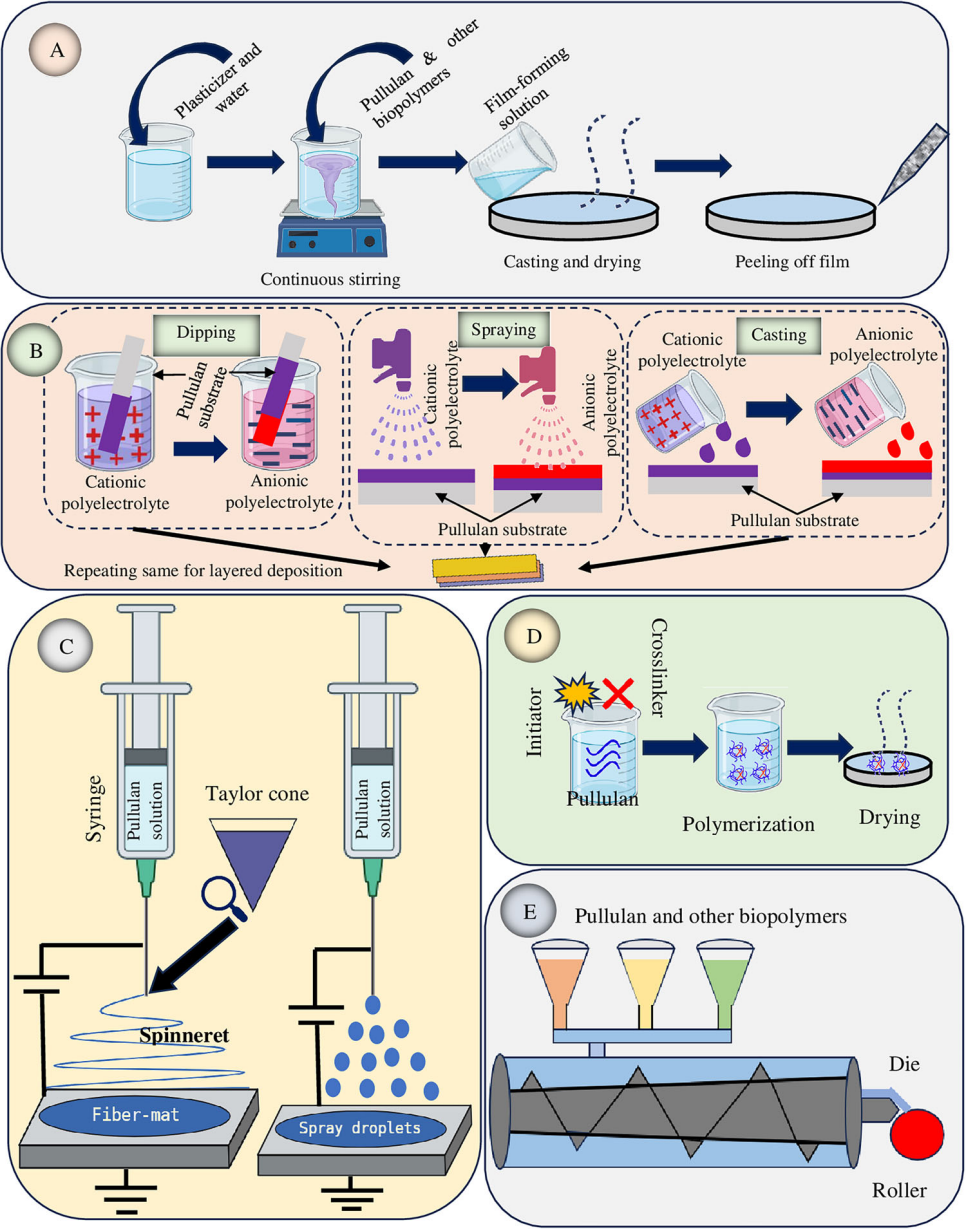
Schematic illustrates different casting techniques used for developing pullulan‐based biocomposites: (A) solution casting, (B) layer‐by‐layer assembly, (C) electrohydrodynamic process, (D) polymer hydrogel, and (E) coextrusion.

Apart from chemical modification of native polymer, pullulan‐based packaging materials could be developed by combining with other polymer types. There have been different strategies employed for obtaining biocomposites of pullulan with desirable properties for food packaging. The scheme given in Figure 3 summarizes various techniques for generating pullulan biocomposites, which are explained in subsequent sections.

### 7.1 Solvent‐Casting

Solvent‐casting is a widely employed technique in laboratory settings for biodegradable film preparation. It typically has three distinct stages, namely, solution preparation, mold casting, and drying. Film‐forming solutions are mixed and cast in a Teflon plate, followed by simple air drying. Air drying may be conducted at room temperature, but depending on the solvent, higher temperatures may be employed for film casting. The molecular arrangement and interactions in the film alter as it dries, contributing to its desired properties (Salević‐Jelić et al. 2023). However, this method is generally limited to laboratory‐scale studies and is not readily applicable for industrial‐scale production, making it more suitable for fundamental research evaluating the characteristics of pullulan‐based films (Othman et al. 2023).

### 7.2 Layer‐by‐Layer Assembly

Instead of mixing multiple polymeric solutions and casting them at once, layer‐by‐layer assembly comprises casting in successive deposition of various film‐forming solutions onto a substrate that provides appropriate barrier and functional qualities (Avila et al. 2023). This technique relies on hydrophobic attraction, hydrogen bonding, or electrostatic attraction between the adjacent layers (Ganjeh et al. 2024). When a negatively charged substrate is submerged in a positively charged solution, electrostatic attraction occurs, attracting the positively charged materials to the substrate's surface. After washing out the surplus solution, the positively charged substrate is immersed in a negatively charged solution, leading to a new surface (Anukiruthika et al. 2020). This procedure must be continued multiple times to create multilayered coatings or films. Carboxymethylation was employed to convert pullulan into a polyanionic biopolymer. This polyanionic biopolymer facilitates interlayer hydrogen and ionic interactions with cationic chitosan, partly modified by butyric acid (Erceg, Aćimović, et al. 2024). As a result, an emulsifying effect is imparted to chitosan, enabling the direct addition of essential oil without the need for surfactant incorporation.

### 7.3 Electrohydrodynamic Process

The electrohydrodynamic method entails the utilization of a high‐voltage electric field to spray (electrospraying) or spin (electrospinning) polymer solutions into particles and fibers, respectively (Charles et al. 2021).

Electrospinning is a highly inventive, robust, and adaptable technology employed in the production of polymeric fibers. It encompasses various techniques, including emulsion and coaxial electrospinning (Silva et al. 2021). It has four major parts, namely, a high‐voltage power source, a capillary tube with a tip, an injection pump, and a metal collector. It uses high‐voltage electrical fields to make charged jets from flexible polymer solutions. These jets dry out to make very thin polymeric fibers (Echegoyen et al. 2017). Due to their nanoscale size, the electrospun fiber mats have a high surface‐to‐volume ratio and porosity, which facilitates a more convoluted route to moisture and oxygen, limiting their permeability and enhancing the encapsulation of bioactive substances in active packaging (Xue et al. 2019). Fiber production depends on many specific solution properties (solvent volatility, viscosity, polymer concentration, surface tension, and electrical conductivity), environmental factors (humidity and temperature), and process conditions (voltage, flow rate, and spinning distance) (Sun et al. 2025).

Electrospray, on the other hand, utilizes an alligator clip on the syringe needle to provide high voltage to the solution (Daassi et al. 2023). Syringe pumps distribute fluid to the blunt needle tip to regulate flow. When an intense electric field is introduced to the syringe fluid, Taylor cone droplets form at the nozzle tip. In the end, electrical forces are stronger than the solution's surface tension, and a straight jet shoots out from the cone's tip. As it approaches a grounded collector, the jet fragment produces nanoparticles by electrospraying, as opposed to nanofibers by electrospinning (Pires et al. 2023). Although electrospinning is a better method for preparing biodegradable films, electrospraying is suitable for coatings. Uniaxial blend electrospinning was employed to develop electrospun nanofibers depending on pullulan and chitin nanofibers. The voltage, receiving distance, and jet speed were set at 26 kV, 15 cm, and 0.1 mm/min, respectively (Duan et al. 2021), whereas pullulan and silver nanoparticle composite nanospheres by electrospraying were fabricated using the applied voltage at 15 kV (Islam et al. 2011).

### 7.4 Polymeric Hydrogel

Polymeric hydrogels possess a three‐dimensional structure and exhibit hydrophilic properties, functioning through physical and chemical bonding mechanisms (Batista et al. 2019). Their potential to encapsulate, entrap, and transport bioactive substances and slow their release is important in the food sector (Narayanan et al. 2021). By enhancing the cross‐link density of the molecules inside the hydrogel structure through interpenetrating networks and physical blending, it serves to fortify and enhance inter‐ and intramolecular connections (Gul et al. 2022). Nanofillers and crosslinking agents strengthen the hydrophilic‐hydrophobic interface of materials, preventing sedimentation or aggregation. In this regard, Rukmanikrishnan and Lee (2021) reported the generation of quaternary ammonium silane (QAS) and montmorillonite (MMT) clay‐reinforced pullulan and agar‐based nanobiocomposites through the hydrogel technique, where MMT clay was used as a nanofiller.

### 7.5 Coextrusion

Extrusion is a cost‐effective industrial procedure for high‐demand applications. Coextrusion involves heating two or more polymers beyond glass transition temperature and extruding them through a specific nozzle to create multilayer films (Agassant and Demay 2022; Apicella et al. 2023). Feeding, stream confluence, melting, and coextrusion are the main stages involved in this process. Coextrusion can be classified into die and feed block types based on how the polymer streams are mixed during film‐forming (Dziadowiec et al. 2023). In die coextrusion, more than one material is introduced into the extruder and melted, followed by mixing at the die's exit, whereas in feed block coextrusion, polymers are fed at a time before the extrusion die, developing a laminated layer of melt stream that is later extruded (Apicella et al. 2023). There have been no studies reporting the coextrusion technique for preparing pullulan‐based biocomposites. Pullulan has a relatively low thermal stability compared to other polymers (Aquinas et al. 2024). Coextrusion involves processing at high temperatures, which may degrade pullulan or alter its functional properties, making it challenging to use without compromising its quality. Therefore, pullulan derivatives or crosslinking techniques may be required to produce an extrusion‐friendly film from pullulan.

## 8 Characteristics of Pullulan/Biopolymer‐Based Composites or Edible Film

Edible films are deemed appropriate for human consumption. Biodegradable composites are generally designed using multiple materials, namely, polysaccharides, lipids, and proteins. Films composed of proteins or polysaccharides often exhibit favorable gas barriers and mechanical characteristics, whereas they demonstrate poor water‐resistive properties (Gan et al. 2024). Within this context, pullulan‐based films have received significant attention in recent years, yet their performance is best appreciated when compared with other common biopolymers. Pullulan films generally exhibit favorable mechanical properties, with high TS (50 MPa) but relatively low EB (3%) and a low WVP (0.8 × 10^−10^ g/(m s Pa)) (Zamanidehyaghoubi et al. 2025). Notably, pullulan films completely degrade in soil within 15 days, underscoring their excellent biodegradability. On the contrary, cassava starch cast films showed low TS (1.71 MPa), but higher flexibility with 50% EB, and high WVP (8 × 10^−10^ g/(m s Pa)) (Gunathilake and Somendrika 2024). However, these films degraded by 75% in neutral organic soil within 10 days. Arrowroot starch film demonstrated moderate TS (10 MPa) and excellent extensibility (EB ∼ 200%) and achieved 83.47% weight loss after 21 days of soil burial (Mathew et al. 2025). Unlike pullulan films, watermelon rind‐derived pectin films possessed inherent functional activity, with a total phenolic content (TPC) of 16 mg GAE/100 g and DPPH scavenging activity of 12%, and disintegrated completely in soil within 12 days (Hasan et al. 2025). Sodium alginate films displayed moderate TS (4.75 MPa), good flexibility (EB ∼ 51%), moderate WVP, and full biodegradation after 14 days (Ruan et al. 2024). Chitosan films outperform pullulan in antibacterial and antioxidant functionality but are slower to degrade (50 days) (Wu et al. 2025). They also exhibited low water solubility (10%) and a high water contact angle (154°), reflecting their hydrophobic character. Similar to pullulan films, gelatin films revealed high TS (61 MPa) and low EB (10%) (Lunar et al. 2025). They expressed a hydrophilic nature with a low water contact angle of 55° and a low WVP of 3.5 × 10^−10^ g/(m s Pa) and degraded by 80% within 5 days. In contrast, films consisting of lipids, such as waxes, provide favorable WVB characteristics (Motelica et al. 2020). However, they exhibit limited flexibility and minimal resilience to mechanical stress, along with inadequate stability against oxidative rancidity. But, in combination, they interact physically and/or chemically and may result in improved features of films depending on the compatibility of selected polymers (Wu et al. 2024).

Different blends of pullulan‐based edible films with plasticizers and techniques for preparing these biocomposites are presented in Table 2. The presence of plasticizers modifies the intermolecular interactions within polymer chains, resulting in an augmentation of the intermolecular free space volumes. Addition of glycerol or sorbitol in acacia gum, pectin, and pullulan‐based edible blend films has resulted in an increase in water solubility of film, improving its degradation (Suresh et al. 2022). They reported that high MW of plasticizer, sorbitol, can reduce hydrophilicity in film as it effectively preserves moisture absorption through the formation of intermolecular hydrogen bonds within its structure. Adding soybean polysaccharides to pullulan solutions makes them convenient for packaging photosensitive powdered foods, as this modification improved optical barrier property (*p* < 0.05) in the UV region (200–400 nm) (Zhao et al. 2024). However, OP and WVP of the films increased owing to the foaming characteristics of soybean polysaccharides that lowered the density of the blend film. Furthermore, introduction of β‐glucan raised the TS by 384% and lowered the OP by 93.3% due to improvement of β‐sheet content, development of two glycosylated proteins, and intermolecular hydrogen bonds (Chang et al. 2019). The biocomposites alone cannot alter the hydrophilic nature of the film, which is required for packaging perishable foods. Therefore, multi‐frequency power ultrasound‐assisted tea polyphenol‐loaded pullulan–trehalose film was successfully used for preserving fresh‐cut pears and apples (Kang et al. 2023). The application of ultrasonic power effectively governs cavitation, hence facilitating physical and sonochemical phenomena, inducing molecular interactions between polyphenols and pullulan molecules, leading to a significant reduction of WVP to 3.49 × 10^−12^ g/(cm s Pa). Similarly, Jia et al. (2022) prepared pullulan–pea protein isolate electrospun nanofiber films, thermally cross‐linked through Maillard reaction by adding glucose. Cross‐linking by heat successfully enhanced hydrophobicity (water contact angle >100°), thermal stability, and barrier properties, as major bonding occurred between the carbonyl group of glucose and the amino group of pea protein isolate. Not only the Maillard reaction but also the Schiff base can enhance the physicochemical characteristics of the film. In this context, Qin et al. (2020) added cinnamaldehyde as a crosslinking agent for making Schiff base and produced electrospun pullulan/chitosan biocomposite films. It increased the water contact angle to 113°, reduced WVP to 1.60 × 10^−12^ g/(cm s Pa), elastic modulus from 344.3 to 1150.9 MPa, and TS from 11.36 to 22.55 MPa, and EB from 4.53% to 2.83%. In another study, Gan et al. (2023) used a two‐step solution casting method to produce an organic bilayer film composed of chitosan and bacterial cellulose, reinforced with a protective layer of pullulan. It was designed specifically to store high‐fat foods by reducing OP and WVP of the film. Although these studies point toward improving pullulan‐based materials to satisfy basic needs of packaging, there has been progress in preparing active coatings or packaging materials through functionalization of the film with bioactive molecules.

**TABLE 2:** Characteristics of pullulan‐based biocomposites using different polymers, plasticizers, and fabrication techniques.

Food and applied conditions	Matrices (pullulan +)	Plasticizers	Techniques	Mechanical properties	Barrier properties	Hydrophilicity	Solubility	Thermal properties	Optical properties	Functional properties	Applications	References
Peptide powder; heat‐sealed bags placed into beaker containing water at 65°C	Soybean polysaccharide	Glycerol	Solution casting	TS: 34.24–35.79 MPa	Poor barrier property as OP and WVP increased	Increased water contact angle but <90°, indicating hydrophilic nature	Decrease in dissolution time	Increase in melt transition temperature of pullulan (70°C) to 83°C in composite	UV barrier improved but slightly resisted visible light	ABTS: Significantly increased from 0.72% to 16.67%	Heat‐sealing packaging promotes no breakage/leakage, but color turned yellow	Zhao et al. (2024)
				EB: 4.52% to 36.70%								
—	Chitosan and bacterial cellulose	Glycerol	Layer‐by‐layer	Improved TS and EB	Lowered OP and WVP	—	Water sensitivity decreased	Increased stability	Improved UV‐blocking	Increased antioxidant activity	—	Gan et al. (2023)
Apple and pear; 25°C for 4 days	Trehalose and carboxymethyl cellulose	Glycerol	Casting assisted by ultrasound	TS and EB increased by 66.78% and 24.35%	WVP and OP significantly (*p* < 0.05) reduced after ultrasound	—	—	Improved due to ultrasound	Superior UV light screening	Excellent antibacterial properties	Prevent moisture loss and inhibit oxygen penetration	Kang et al. (2023)
Cherry; 25°C for 9 days	Arginine‐carboxylated pullulan	Glycerin	Solution casting	—	Water vapor transmittance lowered by 37%	—	Solubility decreased by 14%	—	Transmittance increased by 39%	Minimum inhibitory concentration 55 and 50 mg/mL against *Escherichia coli* and *Staphylococcus aureus*	Retained appearance after 9 days, reduced weight loss by 29%, and increased hardness, TSS, and TA by 50%, 28%, and 6.8%, respectively, after 9th day	Zhang et al. (2023)
—	Pea protein isolate	Thermal crosslinking by adding glucose	Electrospinning	—	OB and WVB properties improved	Water contact angle: 58.6°–104.5° (>90°)	Reduced with the glucose	Stability increased	—	—	—	Jia et al. (2022)
Fish liver oil; heat‐sealed bag at 40°C for 9 days	Gelatin, sodium alginate, and sodium tripolyphosphate	Glycerol	Solution casting	TS lowered, but EB improved	OP lowered by 15%	—	No significant difference (*p* < 0.5)	Stability increased	UV and IR barrier improved	Antioxidant activity improved	Peroxide, anisidine, and malondialdehyde values lowered at Day 9	Li et al. (2022)
Ivy gourd; 27°C and 70% RH	Gum acacia and pectin	Sorbitol and glycerol	Solution casting	Plasticizer increased EB, but lowered TS	WVP and OP lowered	Contact angle increased to 64°, but less than 90°, signifying hydrophilic nature	Plasticizer increased solubility	—	—	—	Lowered weight loss by 7%, retained 0.35% TA, and increased TPC after 14 days	Suresh et al. (2022)
Mushroom and strawberry; 4°C and 85% RH for 12 days	Konjac glucomannan and crystalline cellulose (MCC)	Glycerol	Casting assisted by ultrasound	Significantly increased TS after ultrasound	WVP significantly increased with MCC but decreased after ultrasound	—	Solubility decreased with increasing MCC	—	Ultrasound lowered transmittance from 88.33% to 78.7%	DPPH decreased with increase of MCC under ultrasound	Reduced weight and TSS loss	Zhou et al. (2022)
				EAB decreased with MCC								
—	Chitosan	Thermal crosslinked, cinnamaldehyde crosslink (Schiff base)	Electrospinning	TS: 11.36–22.55 MPa	WVP and OP decreased for both crosslinking	Hydrophilic for thermally crosslinked; cinnamaldehyde crosslinked is hydrophobic	Solubility decreased from 83.65% to 43.85%	Onset degradation temperature of crosslinked films increased	—	—	—	Qin et al. (2020)
				EAB: 4.53%–2.83%								
—	β‐Glucan	Glycerol	Casting	TS: 48.44 MPa	WVP: No significant difference (*p* < 0.5)	—	Dissolution time increased	Endothermic peak at 135°C, showing thermally stable film	—	—	—	Chang et al. (2019)
				EB: 79.74%	OP: Significantly (*p* < 0.5) reduced							

## 9 Current Applications of Pullulan as a Biocomposite in Functional Food Packaging

This section explores pullulan's applications in edible coatings, active packaging through direct incorporation and encapsulation of bioactive substances, and intelligent packaging systems.

### 9.1 Pullulan‐Based Edible Coating

A variety of biodegradable polymers, commonly referred to as “microbial gums,” are synthesized extracellularly during the growth of microorganisms, including bacteria, fungi, and yeasts, using substrates like glucose, sucrose, starch hydrolysates, molasses, whey, lignocellulosic biomass, and other agro‐industrial by‐products (Martínez‐Burgos et al. 2024). Despite their wide application in the food industry as base materials for developing edible coatings and films (Alizadeh‐Sani et al. 2019), carbohydrate‐based microbial gums are also widely explored in the pharmaceutical sector (Mohammadinejad et al. 2020; Koyyada and Orsu 2021). Typical examples include pullulan, levan, xanthan, gellan, and curdlan, which can be produced through controlled microbial fermentation, ensuring consistent quality and scalability independent of seasonal or geographic variability, an advantage over plant‐derived polysaccharides, such as starch, cellulose, and pectin (Sen et al. 2025). Microbial gum‐based edible coatings are inherently hydrophilic due to the strong interaction of polysaccharides with water, which affects their moisture content and WVP (Nehra et al. 2023). Nevertheless, they provide outstanding barrier properties against gases such as oxygen and carbon dioxide, as well as lipids, making them highly suitable for diverse food packaging applications (Zikmanis et al. 2021).

Pullulan exhibits exceptional characteristics as a polysaccharide for edible coatings aimed at enhancing the quality and shelf life of food (Bhari et al. 2025). Edible coating functions as a semi‐permeable barrier against moisture, O_2_ and CO_2_ (Payal et al. 2025; Wibowo et al. 2024). During storage of fruits, for example, total soluble solids (TSS) rise because of the conversion of starch into sugar (Gammage and Marangoni 2025; Lieu et al. 2025). Alongside, softening of fruits results from the degradation of insoluble protopectin and pectin, which play a role in maintaining structural rigidity. Furthermore, antioxidant content and TPC decrease due to the free radical formation and breakdown of cellular structure, respectively, during ripening (Pillai et al. 2024). Evidently, coating over food suppresses respiration, slows dehydration, retains volatile content, improves textural qualities, and reduces spoilage (Ali et al. 2025; Aggarwal et al. 2025). Diverse techniques, including immersing, spraying, brushing, wrapping, foaming, and panning, are employed (Miranda et al. 2024). An edible coating of acacia gum, pectin, and pullulan on ivy guard was reported to effectively reduce free radicals of oxygen and hydrogen peroxide gas, as compared to the control group, and was found to possess significantly higher TPC (*p* < 0.05) (Suresh et al. 2022). Titratable acidity (TA) in most fruits, due to the presence of organic acid, decreases during ripening. Authors claimed retarding the ripening of ivy guard by controlling the decreasing trend of TA during storage. Rastali and Chakkarakeli bananas were coated with pullulan through dipping, brushing, and sprinkling (Ganduri 2020). Among the methods, the dipping method with a 10% concentration prevented weight loss more effectively after storing at 25°C ± 1°C for 20 days. The research conducted by Kumar et al. (2020) examined the effects of an edible coating made from a blend of pullulan and chitosan, enriched with pomegranate peel extract, on litchi during an 18‐day storage period at both cold (4°C ± 3°C, RH 90% to 95%) and room temperature (23°C ± 3°C, RH 40% to 45%). The lowest increase in TSS was observed in 4°C‐treated litchi compared to room temperature storage. Even the total phenolic and flavonoid contents of litchi at both room and cold temperatures were maintained for 18 days in the coated fruits. Bilayer coating systems of pullulan–gelatine or pullulan–chitosan using combinations of curry plant and lemongrass hydrolats were revealed to be effective against artificial contamination before and after application in sliced cheese (Erceg, Šovljanski, et al. 2024). Roasted peanuts display a high peroxide value (PV) owing to lipid oxidation. As shown by Priyadarshi et al. (2022), grape seed extract‐loaded pullulan–pectin coating could prevent the oxidation of peanut during storage. Grape seed extract possesses strong antioxidant activity in blend coating due to the free radical scavenging property of polyphenolic components. After 30 days of storage, the PV of roasted and raw uncoated peanuts was 70.8 and 0.57 meq/kg oil, respectively. However, the PV of coated peanuts decreased to 53.6 and 0.32 meq/kg oil for roasted and raw peanuts, respectively, causing a 75% reduction in PV and delay in rancidity. Similarly, the shelf life of the strawberry was extended by 24 days at 4°C by a 50:50 ratio of pullulan and apple pomace pectin and 15% apple pomace phenolic compound (Salimi et al. 2024). In addition to natural bioactives, potassium metabisulfite and pullulan coating were evidenced to prevent the mycelial growth of *Phomopsis* sp., *Botryosphaeria dothidea*, *Alternaria* sp., and *Diaporthe* sp., the main microbes for rotting of kiwifruit (Tian et al. 2023).

Apart from vegetables and fruits, the applicability of pullulan blends has also been tested as a dip coating on fish and meat products. Zhu et al. (2024) extracted melleolide from *Floccularia luteovirens* and developed coating with pullulan to improve the shelf life and quality of Pacific white shrimp during refrigerated storage. The study revealed that melleolide exhibited potent antibacterial activity, forming clear inhibition zones against *Bacillus cereus* (12.8 mm), *S. aureus* (11.8 mm), and *E. coli* (9.5 mm). Beyond its antimicrobial effects, melleolide also significantly inhibited polyphenol oxidase, the enzyme responsible for melanosis in shrimp (Sae‐leaw and Benjakul 2019), achieving a maximum inhibition rate of 73.52% and an IC_50_ of 6.27%, likely through reversible, noncovalent binding. During storage, the total volatile base nitrogen (TVB‐N) content in coated shrimp remained well below the spoilage threshold of 30 mg/100 g, reaching only 16.1 mg/100 g on Day 6, in contrast to 32.43 mg/100 g in the untreated control. Furthermore, the treated shrimp maintained aerobic plate counts below 5.7 log CFU/g and thiobarbituric acid reactive substances (TBARS) values under the critical limit of 1 mg MDA/kg for a significantly extended period, whereas the control group reached spoilage by Day 6. Similarly, Li et al. (2021) assessed the shelf life of sea bass fillet applied with sodium carboxymethylcellulose, ε‐polylysine hydrochloride, and gallic acid into pullulan. They reported that gallic acid with pullulan had a cumulative effect on enhancing the protective effect of pullulan and carboxymethylcellulose, as they suppress microbial growth, protein degradation, lipid oxidation, and control texture properties.

Despite the widespread use of pullulan‐based edible coatings, there remains a pressing need to develop pullulan‐based active packaging films that can extend functionality and enhance food preservation. The following sections examine studies conducted on the incorporation of active substances into packaging film, either through direct addition or via encapsulation techniques.

### 9.2 Direct Incorporation of Active Substances in Pullulan‐Based Active Packaging

The concept of active packaging targets to achieve additional functionalities where food, package, and surrounding environment synergistically enhance safety and sensory attributes of food (Ahmed et al. 2022). The physicochemical and functional characteristics of pullulan‐based packaging may be improved by either modifying the surface of pullulan or blending it with other biopolymers. Aside from these, antimicrobials are introduced into the matrix of pullulan‐based films to impart antifungal or antibacterial action, thus reducing food oxidation and the proliferation of spoilage microorganisms. These antimicrobials might be organic (viz., plant extracts and essential oils, salts, acids, and antibacterial enzymes) or inorganic (e.g., silver nanomaterials) (Martins et al. 2024). There have been many reports on the direct incorporation of antimicrobials into pullulan‐based films for improving functionality. The antimicrobial mechanisms of pullulan‐based active films largely depend on the type of incorporated antimicrobial substances. For example, essential oils and polyphenols act primarily through disruption of microbial cell membranes, leakage of intracellular contents, and oxidative stress induction (Maryoris et al. 2020), whereas metal‐oxide nanoparticles often exert photocatalytic or electrostatic effects that damage microbial proteins and DNA (Shkodenko and Kassirov 2020; Mukherjee et al. 2023). Moreover, cationic substances exert their antimicrobial action by strongly interacting with the negatively charged microbial cell membranes, disrupting their structural integrity and ultimately causing cell lysis (Zhou et al. 2023).

Broiler meat is very susceptible to oxidative rancidity at room temperature during storage. But pullulan‐mediated silver nanoparticles and curcumin‐added pullulan active packaging were found to increase oxidative stability as evidenced by lower concentrations of malondialdehydes (3.07 mg/kg meat), higher water‐holding capacity (87.22%), and expressible fluids (59.17%) and lower drip losses (2%) after 7 days of refrigerated storage (Khan et al. 2022). Green carbon dots synthesized from galla chinensis waste led to 6.25% enhancement in TS of the pullulan film along with reduction of WVP (1.47 × 10^−12^ g/(cm s Pa)). Herein, the OH groups on carbon dots were proposed to promote the development of hydrogen bonds with pullulan, resulting in a decrease in the accessible free volume and an improvement in gas sealing. Additionally, this interaction restricts the mobility of water vapor particles (Tang et al. 2024). Modification of pullulan with carbon dots also increased the antioxidant activity of the film because of the occurrence of several functional groups such as hydroxyl, carboxyl, and amino groups. In another study, clove essential oil incorporated chitosan–ZnO hybrid nanoparticles into pullulan–chitosan film and was found to enhance TS (39.82%), WVB (84.64%), OB (57.66%), and UV blocking ability stemming from generation of more intermolecular hydrogen bonding and high specific surface area of nanoparticles (Gasti et al. 2022). They further highlighted that the addition of clove essential oil‐loaded chitosan–ZnO nanoparticles into composite film enhanced antioxidant activity due to the presence of free NH_2_ groups in chitosan molecule, electron density located at oxygen of ZnO present in nanoparticles, and inherent antioxidant property of clove essential oil.

Besides studying functional properties, it is equally important to evaluate the release behavior of active substances from pullulan‐based films. In this context, Kowalczyk et al. (2020) investigated the release kinetics of potassium sorbate from pullulan‐based films into aqueous medium for up to 1 h. They observed a burst release immediately after immersion, occurring 50% migration within 1 to 5 min due to excellent water solubility of pullulan. In contrast, incorporation of gelatin into pullulan films slowed the release rate of potassium sorbate by 2.5 times, attributed to the lower swelling capacity and erodibility of gelatin. Among several models tested, including zero‐order, first‐order, Higuchi, Korsmeyer–Peppas, Hixson–Cowell, Baker–Lonsdale, Makoid–Banakar, and Weibull model, the Weibull model provided the best fit, as indicated by the lowest Akaike Information Criterion values, a metric used to compare the relative quality of various statistical models. In another study, Carvalho et al. (2021) examined the migration of indomethacin from pullulan‐graft‐poly(ε‐caprolactone) nanoparticles into phosphate buffer solution at 37°C temperature for 24 h. They reported that pure indomethacin dissolved completely within 1 min, whereas encapsulated formulations exhibited sustained release, with 100% release observed only after 360 min. Notably, Korsmeyer–Peppas model described the release profile of indomethacin with the highest correlation coefficient, indicating that the mechanism followed a non‐Fickian diffusion pathway.

Unlike organic substances, inorganic particles are found to improve physicochemical and functional properties of pullulan‐based film. For instance, nano‐TiO_2_‐integrated pullulan and carboxymethyl cellulose was used for preserving strawberries by significantly maintaining the TA, firmness, vitamin C, and skin color and reducing weight loss of strawberries (Zhang et al. 2021). This active packaging showcased significant antibacterial activity against *E. coli* and *S. aureus* because of photocatalysis action of nano‐TiO_2_. Even WVB and UV barrier properties of nano‐TiO_2_‐integrated pullulan were effectively enhanced. The addition of QAS and MMT clay into agar/pullulan composite revealed excellent mechanical and thermal characteristics (Rukmanikrishnan and Lee 2021). The incorporation of QAS exhibited notable antibacterial efficacy against both gram‐positive and gram‐negative microbes, attributed to the presence of quaternary nitrogen groups. The interaction between hydrophilic side groups of MMT clay and water molecules results in a reduction in the available molecular volume within the polymer matrix and inhibits the mobility of polymer chains. Therefore, it significantly improved WVP (1.585 to 1.10 × 10^−15^ g/(cm Pa s)) of film. In the subsequent sections, we discuss the major techniques to enhance the activity of bioactive substances and their impact on pullulan‐based packaging film.

### 9.3 Role of Encapsulation Techniques in Pullulan‐Based Active Packaging

Encapsulation of bioactives has been regarded as an effective strategy prior to their incorporation into film matrices, particularly in pullulan‐based packaging, where direct addition of such compounds often presents significant challenges (Mukurumbira et al. 2022). Many bioactives, such as essential oils and polyphenols, are volatile and thermosensitive, leading to substantial losses during film fabrication processes like drying or solvent evaporation, ultimately reducing their effectiveness (Westlake et al. 2022). Additionally, undesirable chemical interactions between bioactives and the biopolymer matrix can result in reduced bioactivity; for instance, phenolic compounds may form hydrogen bonds with pullulan's hydroxyl groups, limiting their functional availability (Nilsuwan et al. 2018), whereas flavonoids may undergo oxidation or bind with ions, reducing their antimicrobial or antioxidant properties (Arfat et al. 2015). Another critical issue is the rapid and uncontrolled release of free bioactives from the hydrophilic pullulan matrix during storage, which compromises long‐term functionality. In this regard, Wang et al. (2018) reported a burst release of catechin from chitosan films, with most release occurring within the first 6 h. Moreover, direct incorporation of hydrophobic compounds often leads to phase separation, affecting the homogeneity, transparency, and mechanical integrity of the film. For instance, Chu et al. (2019) observed poor dispersion and significant opacity in pullulan films containing nonencapsulated cinnamon oil, whereas Ranade et al. (2025) found reduced TS in pullulan‐based films with directly added curcumin.

**FIGURE 4:**
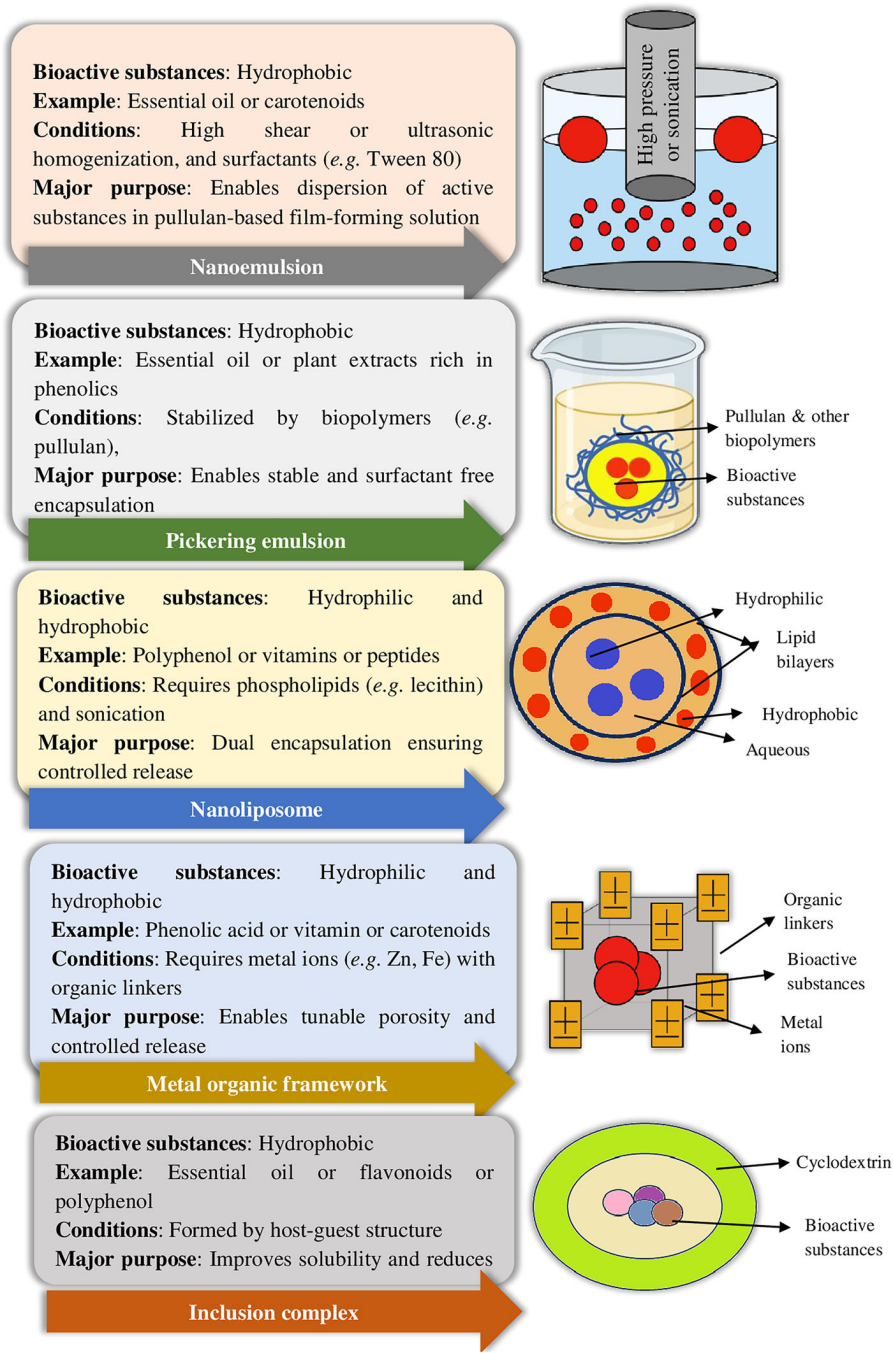
Different stabilization techniques of bioactive compounds for active packaging of pullulan.

These issues highlight the necessity of stabilization techniques such as nanoencapsulation, Pickering emulsions, inclusion complexes, and nanoliposomal systems, which enhance the retention, controlled release, and compatibility of bioactives within pullulan‐based composites. Figure 4 summarizes an overview of the major encapsulation techniques, including nanoemulsion, Pickering emulsion, nanoliposome, metal organic framework (MOF), and inclusion complex, highlighting the nature of bioactive encapsulation, preparation conditions, and functional outcomes for pullulan‐based packaging systems. These strategies not only protect the integrity of sensitive compounds in film during processing and storage but also support sustained functional performance, positioning encapsulation as a vital step in the development of advanced pullulan‐based active packaging materials.

#### 9.3.1 Encapsulation in Cyclodextrin (CD)

CD, a cyclic oligosaccharide, consists of glucose molecules connected by α‐(1→4)‐glycosidic linkages (Patino Vidal et al. 2022). Standard CD utilized in packaging includes α‐CD, β‐CD, and γ‐CD, which are composed of six, seven, and eight glucose units, respectively. The encapsulation of hydrophobic bioactive substances can be achieved by CDs, owing to their stable hydrophobicity in both liquid and solid conformations, biocompatibility, and lower cost (Maniam et al. 2022). In this regard, the incorporation of carvacrol and γ‐CD inclusion complexes into gelatin and pullulan composite nanofibers led to a 67.84% carvacrol retention after 2 months at room temperature, compared to 57.63% in nonencapsulated nanofibers, enhancing both stability and antibacterial performance (Ertan et al. 2023). Similarly, encapsulated essential oil‐loaded pullulan nanofibers preserved 74%–77% of volatile essential oils like geraniol and linalool, compared to only 15%–23% retention in nonencapsulated nanofibers. This also enhanced their thermal stability and long‐term storage stability, shifting the volatilization range from 119–139°C to 239–292°C and retaining 48%–64% of the essential oil content after 4 weeks. Furthermore, these nanofibers exhibited superior antibacterial activity against both Gram‐positive (*S. aureus*) and Gram‐negative (*E. coli*) bacteria (Celebioglu et al. 2024).

#### 9.3.2 Nanoemulsion

In an emulsion, lipid‐based hydrophobic compounds are dispersed in water as droplets. Surfactants minimize interfacial tension by adsorbing to the lipid–water contact, stabilizing the emulsion (Zhang et al. 2022). On the basis of their size, the emulsion is split into microemulsion (200 nm–200 µm) and nanoemulsion (<200 nm) (Almasi et al. 2021). Nanoemulsions have better stability due to a high surface area ratio than crude emulsions, minimizing rapid release from self‐accumulation and improving bioavailability (Pandey et al. 2022). Furthermore, nanoemulsions exhibit a diminutive droplet, enhancing the preservation of the initially arranged order inside the film matrix. Consequently, this characteristic mitigates adverse effects on the performance of the film (Ahari and Naeimabadi 2021). Zhang et al. (2025) developed pullulan‐based antimicrobial packaging films incorporating thymol and waxy maize starch nanoemulsions, using a high‐pressure microfluidization technique, to enhance the storage quality of strawberries. The nanoemulsion exhibited a droplet size of 127.9 nm and a zeta potential of −32.4 mV, indicating high dispersion stability. Films also demonstrated strong antibacterial efficacy, particularly against *S. aureus*, attributed to the sustained release of thymol. Similarly, Yao et al. (2025) developed pullulan and gellan gum composite films by incorporating astaxanthin nanoemulsions, which exhibited droplet sizes between 167.70 and 351.03 nm and retained 95.37% of astaxanthin after 7 days of storage at 25°C. These films demonstrated strong antioxidant activity with an IC_50_ value of 14.87 µg/mL and effective antibacterial action against *E. coli* and *S. aureus*. Further, the researcher applied the polymeric coating on strawberries, reducing weight loss to 12.48%, preserving 7.4% TSS, 0.75% ascorbic acid, and 36.45 mg/100 g TA, and extending shelf life from 12 to 18 days.

#### 9.3.3 Pickering Emulsion

Standard emulsions, such as coarse and nanoemulsions, employ surfactants, such as Tween 80, to effectively disperse the oil phase droplets within the continuous water phase. In the Pickering emulsion, the stabilization of emulsions is achieved through the utilization of solid particles, unlike surfactants (Cheng et al. 2024). In contrast to conventional emulsions, the solid layer serves as a mechanical barrier, preventing coalescence and leading to enhanced stability caused by steric repulsion and irreversible adsorption (Brito et al. 2022). This solid layer also serves as a carrier and a shield for bioactive antibacterial components against oxidation. Shen et al. (2021) stabilized clove essential oil by both nano and Pickering emulsions into inulin and whey protein isolate mixture for incorporating into gelatin–pullulan film. The droplet diameters of nanoemulsions and Pickering emulsions filled with clove essential oil were 15.93 and 266.9 nm, respectively, confirming the stability and efficacy of emulsions. In contrast to nanoemulsion, Pickering emulsion loaded with clove revealed prolonged‐release behavior in the composite matrix, as it was controlled by the inulin and whey protein isolate complex and lowered the release rate. Similarly, Ding et al. (2023) developed a pullulan–gelatin composite film incorporating Pickering emulsion of eugenol to enhance the preservation of chilled beef. Pickering emulsion exhibited a droplet size of 296.0 nm, a polydispersity index of 0.457, and a zeta potential of 20.1 mV, indicating good emulsion stability. The researchers reported that 0.6% Pickering emulsion significantly reduced WVP of film to 6.04 × 10^−9^ g/(cm Pa s). They discovered that application of these films on chilled beef effectively slowed increases in pH, TVB‐N, and TBARS over 15 days of storage, compared to control samples. Notably, the total viable count in beef wrapped with 0.6% Pickering emulsion film was 8.34 log CFU/g on Day 11, lower than the control's 9.43 log CFU/g, indicating significant microbial growth inhibition.

#### 9.3.4 Nanoliposomes

Nanoliposomes consist of phospholipid bilayer membranes of food‐grade that encapsulate an aqueous medium (Bondu and Yen 2022). In this regard, Najafi et al. (2021) developed pullulan‐based edible films incorporating saffron extract encapsulated within nanoliposomes to enhance the stability and functionality of saffron's bioactive components. They prepared nanoliposomes using rapeseed lecithin through sonication, resulting in nanoparticles with sizes ranging from 118 to 138 nm, zeta potentials between −32.0 and −46.8 mV, and polydispersity indices below 0.3, indicating good stability over 28 days at 4°C. The encapsulation efficiency of crocin, a major antioxidant in saffron, was approximately 30%, and release studies in phosphate‐buffered saline showed that about 70% of crocin was released from nanoliposomes within 5 h, suggesting a sustained release profile. Incorporating nanoliposomes into pullulan films via casting led to a reduction in mechanical resistance and thermal stability compared to pure pullulan films. Although the effect on the WVP was not significant, the integration of nanoliposomes and polymers facilitated the formation of a more condensed structure. Consequently, the passage of O_2_ molecules through the film became challenging. Later again, Najafi et al. (2022) developed electrospun nanofibers composed of pullulan, pea protein isolate, and pectin incorporating echium oil and saffron extract encapsulated within nanoliposomes. FTIR of encapsulated nanofibers revealed that the ratio between peaks at 3010 and 2925 cm^−1^, indicative of the unsaturation degree of essential oil, increased over storage time at 50°C, suggesting superior protection against oxidation. Furthermore, the activation energy for essential oil oxidation was highest (100.8 kJ/mol), implying enhanced oxidative stability.

#### 9.3.5 Metal Organic Framework

The organic ligands (or groups) and metal ions are joined by coordinate bonds to produce MOF (Sultana et al. 2022). It has become a promising porous compound with adjustable features, notably, high specific surface area and porosity (Sultana et al. 2022). Release mechanisms of bioactive compounds can be enhanced through the entrapment within the porous structure of the MOF, facilitating a slow and controlled release (Nilsuwan et al. 2018). Furthermore, MOF particles can neutralize free radicals, decompose superoxide, or quench singlet and triplet oxygen by donating hydrogen or electrons to edge site properties, including –NH_2_, –OH, and –COOH functional groups (Sharanyakanth and Radhakrishnan 2020). Therefore, Hong et al. (2024) incorporated cinnamon essential oil‐loaded MOF into pullulan and gelatin composite films. The authors quantitatively reported essential oil loading capacity in MOF to be 32.78% due to high BET surface area (1476.70 cm^2^/g) and total pore volume (0.89 cm^2^/g) of MOF. They found that essential oil‐loaded MOF enhanced antibacterial activity of MOF against *Salmonella enterica*, *S. aureus*, *Listeria monocytogenes*, and *E. coli*, as evidenced by increase in inhibition zone from 28.57, 26.96, 27.31, and 15.72 to 29.46, 29.03, 32.14, and 26.79 mm, respectively. Similarly, at 500 µg/mL MOF concentration, ABTS radical scavenging activity enhanced from 48.78% to 79.95% after cinnamon oil loading. Furthermore, Mou et al. (2025) incorporated citral and geraniol encapsulated in MOF into composite film of modified chitosan and pullulan. They reported that the film demonstrated excellent mechanical properties, with TS and EB reaching 27.91 MPa and 24.52%, respectively. The film exhibited 19.8 and 21.3 mm inhibition zones against *E. coli* and *S. aureus*, respectively. Furthermore, application of the edible film on fresh peaches effectively preserved postharvest quality by maintaining fruit firmness, limiting weight loss to under 4.5% over 10 days, and significantly inhibiting microbial proliferation, collectively contributing to an extended shelf life and enhanced storage stability.

The addition of both organic and inorganic bioactive compounds into pullulan‐based films has shown remarkable potential in enhancing the functional and physicochemical attributes crucial for active packaging. The details of stabilization techniques and the effects of organic bioactive compounds and inorganic engineered materials in pullulan‐based film are summarized in Table 3. Although direct incorporation methods have proven effective, the encapsulation of bioactive substances significantly improves their stability, controlled release, and compatibility with the pullulan matrix. Among the techniques discussed, nanoemulsions and nanoliposomes offer improved dispersion and bioavailability, whereas Pickering emulsions and MOFs contribute to enhanced oxidative stability and sustained release. CD inclusion complexes and electrospinning further safeguard volatile and thermally sensitive compounds during processing. The reported improvements in TS, WVB, UV resistance, and microbial inhibition validate the applicability of encapsulated systems for real‐world packaging scenarios, especially for perishable and high‐value food products. Encapsulation significantly increases formulation and processing costs due to the need for high‐purity ingredients and specialized infrastructure, such as high‐pressure homogenizers or sonicators, making adoption difficult for small‐ and medium‐scale packaging industries. Therefore, selecting an appropriate encapsulation method should align with the desired release profile, physicochemical compatibility, and processing conditions, ensuring optimal functional performance of pullulan‐based active packaging.

**TABLE 3:** Characteristics of pullulan‐based active and intelligent packaging using different bioactive organic and inorganic substances and their stabilization techniques.

Active packaging with bioactive organic compounds											
Food and applied conditions	Matrices	Active substances	Technology for stabilizing active substances	Water solubility	Mechanical properties	Barrier properties	Thermal stability	Appearance	Antioxidant, antibacterial, or antifungal activity	Applications	References
Chicken; 4°C for 12 days	Pullulan and polyvinyl alcohol	Probiotic bacteria (*Lactobacillus plantarum, Lactobacillus acidophilus, and Lactobacillus rhamnosus*)	Electrospinning	—	TS lowered by 68% and EB increased by 148%	WVP increased	*Lactobacillus plantarum* added nanofiber improved thermal stability	Yellow colored	Highest viability rate and excellent antibacterial efficiency for nanofiber incorporated *Lactobacillus plantarum*	*Lactobacillus plantarum* added nanofiber lowered total aerobic and psychrotrophic count most effectively	Dikmetas et al. (2025)
										Maintained pH below 6.5 after 12 days	
										Total volatile basic nitrogen below 25 mg/100 g after 12 days	
Bread; 25°C and 50% RH for 12 days	Chitosan and pullulan	Tangerine peel‐derived carbon dots	Casting	Water solubility increased	TS and EB increased by 23% and 117%, respectively	UV‐barrier and WVB property improved	—	Light yellow colored	DPPH scavenging: 11.4%–62.6%	No mold growth and 17.9% weight loss and less than 3.7 log CFU/g total viable colony count after 12 days	Sul et al. (2025)
									ABTS scavenging: 24.2%–91.6%		
									*Listeria monocytogenes* and *Escherichia coli* growth lowered by 8.9 and 8.4 CFU/mL, respectively		
Strawberries; 25°C and 4°C for 12 days	Pullulan	Thymol essential oil	Nanoemulsion	Completely dissolved	—	Poor water barrier property	Peak temperature of decomposition lowered	—	Inhibition zone against *Staphylococcus aureus* and *E. coli* −11.77 and 13.33 mm, respectively	No wrinkling observed before 8th day at 4°C	Zhang et al. (2025)
										Weight loss and respiration rate delayed, firmness reduced after 6th day, viable count −1.16 log CFU/g after Day 8 at 25°C	
Beef meat; 10°C for 16 days	Pullulan and gelatin	Cinnamon essential oil	MOF	Decreased significantly (*p* < 0.05)	TS and EB did not change significantly	UV‐barrier and WVB property improved	Thermal stability improved	Greenish tint	ABTS scavenging: 19.22%–98.16%	Reduce weight loss, acceptable pH up to 16 days	Hong et al. (2024)
						Transparency decreased			Significant antibacterial activity	Inhibit meat aging and texture loss	
Fresh‐cut pepper and kiwi; 25°C for 6 days	Pullulan and curdlan	Polylysine	Casting	—	TS and EB increased by 64% and 100%, respectively	Hydrophobic	Lowered weight loss rate, signifying thermal stability	Turbidity increased	Inhibited the growth of *E. coli* and *S. aureus*	Slowed decrease of weight loss rate, hardness, and TSS	Liang et al. (2024)
						WVP and OP lowered				Aerobic plate count −6.98 and 5.44 log CFU/g in pepper and kiwi after 6 days	
						UV‐shielding ability improved					
Chicken breast and tofu inoculated with 100 µL of *L. monocytogenes* and *E. coli* (105 CFU/mL) and stored at 10°C	Pullulan and cellulose nanofibers	Zn‐doped avocado peel‐derived carbon dots	Casting	—	TS and EB increased by 46% and 69%	Visible and UV blocking ability improved	—	Yellowish‐brown	5% carbon dots added composite exhibited 100% and 68% ABTS and DPPH scavenging, respectively, and completely restricted *L. monocytogenes* and *E. coli*	Chicken color darkened, but no change in tofu appearance during storage	Riahi et al. (2024)
						Hydrophilic				Lowered thiobarbituric acid reactive substances, pH, and inoculum growth for chicken and tofu	
Strawberry; ambient temperature for 10 days	Pullulan	*Galla chinensis* waste‐derived carbon dots	Casting	Water solubility increased	TS decreased by 24% and EB increased by 43%	Hydrophilic	No significant improvement	Redness and yellowness increased	ABTS: 0.73%–93.61%	6.65% weight loss, 28% decay rate and firmness increased	Tang et al. (2024)
						WVP lowered by 59%			DPPH: 0.15%–86.30%		
						UV‐blocking ability improved			Low concentration of carbon dots exhibited more antibacterial effect on *S. aureus* compared to *E. coli*		
Fish oil; 40°C for 16 days	Pullulan and gelatin	Carvacrol and cyclodextrin	Inclusion complex	—	—	Lower WVP	Thermal stability improved	Retains color	Significant antibacterial activity; strong antioxidant activity	Lowered peroxide value after 8 days	Ertan et al. (2023)
Fresh ham slices; 4°C for 7 days	Pullulan, zein and paper	Rockrose essential oil	Laminating at 17 bar and 100°C	—	TS decreased and EB increased	Low transparency	Thermal stability increased	Slightly yellow colored	Antibacterial activity against *S. aureus* and *L. monocytogenes*	Lowered weight loss and retained texture after 7 days	Bilohan et al. (2022)
						OP lowered					
Chicken meat; 8°C for 6 days	Chitosan and pullulan	Clove essential oil; chitosan–ZnO hybrid nanoparticles	Sol–gel technique	14.98% water solubility increased	EAB reduced and TS increased	UV blocking enhanced by 6.16%	—	—	High antioxidant capacity	Increase in pH of chicken meat packed in nanocomposite film was delayed	Gasti et al. (2022)
						Significantly (*p* ≤ 0.05) reduced WVP and OP			Superficial bactericidal effect		
—	Pullulan and glycerol	Propolis	Casting	Solubility decreased	TS and EB decreased	Good barrier properties against UV	—	Yellow‐orange color	Significant antifungal activity (*p* < 0.05)	—	Gniewosz et al. (2022)
—	Pea protein isolate, pullulan, and glucose for Maillard reaction	Allicin	Electrospinning	Water solubility decreased	—	WVP declined	Improved the thermal stability	—	Antimicrobial activity enhanced at 15% allicin loading	—	Jia et al. (2022)
									Electrospinning effectively protects biological activity of allicin under heating		
—	Pullulan nanofiber	Zanthoxylum bungeanum essential oil and β‐cyclodextrin	Precipitation and inclusion complex	—	Elastic modulus: 82.03–57.38 MPa	—	Essential oil‐loaded inclusion complex enhanced the thermal stability	—	Inhibition rate against *E. coli* and *S. aureus* increased	Total release of essential oil in nanofiber films was about 15% which was significantly less within 30 days	Qin et al. (2022)
					TS: 2.92–2.09 MPa				Antioxidant activity increased		
					EB: 26.21%–3.83%						
—	Pullulan	Saffron extract into lecithin	Nanoliposome	—	TS and EAB decreased	WVP didn't significantly change	Thermal stability lowered	—	Benefits for health‐allegation from saffron extract	—	Najafi et al. (2021)
						But OP reduced					
—	Pullulan, gelatin, and glycerin	Clove essential oil	Nanoemulsion in tween 80 and Pickering emulsion in whey protein isolate‐insulin mixture	Moisture absorption % increased but reduced after adding Pickering emulsion	TS and EB increased	WVP lowered	Thermal stability lowered	Yellowness index increased	Increased antibacterial activity	—	Shen et al. (2021)
									Enhanced DPPH scavenging activity		

**Active packaging with inorganic substances**											
Indian gooseberry; 22°C and 40% RH for 8 days	Pullulan, zein, and chitosan	TiO_2_ nanoparticles	Coating and casting	Water solubility increased slightly	TS decreased by 50%	Superior UV–vis blocking ability	Degradation temperature for 50% weight loss lowered	—	Significant DPPH antioxidant activity and antifungal activity against *Cladosporium*, *Penicillium*, and *Aspergillus*	Rapid browning and yellow discoloration for coating treatment after 2 days	Liu et al. (2025)
						WVP increased by 14%				Browning, wrinkling and decay after Day 8 for film treatment	
						Opacity increased				CO_2_ retention of fruit with coating −2.33 times higher than those with film	
Tofu; room temperature for 3 days	Pullulan and chitosan	Montmorillonite clay	Casting	Moisture adsorption and water solubility lowered	TS increased by 269% and EB lowered by 43%	Water contact angle −90°	—	—	Strong antibacterial activity against *E. coli* and *B. subtilis*	Delayed yellowing of tofu	Madihalli et al. (2024)
						WVP and OP lowered by 34% and 64%, respectively			DPPH scavenging: 26%–38%	16.67% weight loss, 5.83 pH after 72 h	
						Lowered UV and visible light transmittance				Protein content lowered from 15.34 to 12.89 g/100 g and 14.74 g/100 g for unwrapped and clay added composite, respectively	
—	Pullulan, chitosan and glycerol	Chitosan modified halloysite (CHT) nanotube and rutin	Casting	—	TS increased by 20% with 2% CHT but rigidity was not significantly changed	Combined addition of CHT and rutin revealed significant WVB properties	Increase in thermal stability due to crosslinking by CHT	Brightness slightly increased by adding CMT	Increased antimicrobial activity due to surface modification of halloysite with chitosan	—	Roy and Rhim (2021)
—	Pullulan, agar, and glycerin	Montmorillonite clay and quaternary ammonium silane	Casting	—	TS increased and EB decreased	WVP decreased	Slightly improved	Transmittance decreased	More active against *Cronobacter sakazakii* and least active against *Bacillus cereus*	—	Rukmanikrishnan and Lee (2021)
Strawberry; 4°C and 80% RH for 10 days	Pullulan and carboxymethyl cellulose	TiO_2_ nanoparticles	Casting	Water solubility decreased	TS first increased	WVB, UV, and light barrier properties significantly improved	Thermal stability enhanced	—	Superior antibacterial activity	Controlled TA, firmness, vitamin C, and skin color; decreased weight loss	Zhang et al. (2021)
					EB decreased						
—	Pullulan, pectin and glycerol	Silver nanoparticles	Casting	—	TS and EAB decreased	Transmittance decreased	—	Significantly (*p* < 0.05) decreased the *L*‐value	Enhanced antibacterial activity	—	Lee et al. (2019)
						WVP significantly increased					
**Intelligent packaging**											
**Food and applied conditions**	**Matrices**	**Halochromic indicators**	**Techniques**	**Water solubility**	**Mechanical properties**	**Barrier properties**	**Thermal stability**	**pH‐response**	**Antioxidant, antibacterial, or antifungal activity**	**Applications**	**References**
Shrimp; 4°C for 4 days	Dialdehyde pullulan and chitosan	Anthocyanin from black rice	pH‐sensitive	—	Anthocyanin decreased TS but dialdehyde pullulan increased TS	95% UV blocking ability	Anthocyanin reduced thermal stability	Purple at pH < 6, light purple at pH 6, bluish‐violet at pH > 6	—	Film color changed to dark purple and bluish violet on Days 2 and 4, respectively	Xu et al. (2025)
					Anthocyanin and dialdehyde pullulan increased EB		Dialdehyde pullulan increased thermal stability			Total volatile basic nitrogen exceeded 20 mg/100 g after Day 3	
							WVP lowered				
Milk; 25°C for 96 h	Pullulan and sodium alginate	Anthocyanin from cowberry	pH‐sensitive	Lowered water solubility	TS increased, but EB lowered	Superior UV blocking ability	Decomposition rate lowered	Red at pH 2.0–4.0, pink at pH 5–7, yellow–green at pH 8–12	DPPH and ABTS scavenging −13.22% to 24.33% and 15.62%–22.63%, respectively	Film color changed from dark brown to brown and yellow after 48 and 96 h, respectively	Yu et al. (2025)
						WVP and OP lowered by 33% and 51%, respectively			Inhibition zone against *S. aureus* and *E. coli* −13.79 and 11.68 mm, respectively	Fresh milk pH lowered from 6.79 to 5.58 and 4.98 after 48 and 96 h, respectively	
Fish and shrimp; 25°C	Pullulan, sodium alginate and glycerol	Anthocyanin from blueberry	pH‐sensitive	Lowered water solubility	—	WVP decreased	High thermal stability	Red at pH 2.0, lavender at pH 7.0, bule–purple at pH 8.0–10.0	Stronger antimicrobial efficacy	Color changed from pink to darkish pale grey and dark grey during fish and shrimp spoiling	Rashid et al. (2024)
Sea bass meat; 25°C and 65% RH	Pullulan, chitosan and chitin nanofiber	Red cabbage anthocyanin	pH‐sensitive	—	TS: 36.40–42.29 MPa	WVP: 6.25 × 10^−5^ g/m/Pa/day	Improved thermal stability	Red at pH 2.0–4.0, blue at pH 8.0–10.0, and green at pH 11.0–12.0	Radical scavenging ability: 20.87%–52.06%	Blue color of film changed to blue–green and yellow–green after 48 and 96 h	Wu et al. (2022)
					EB decreases	UV–vis light barrier property improved			Excellent antibacterial activity	Yellowness of the film increased	
*Plectorhynchus cinctus*; room temperature for 72 h	Pullulan and chitin nanofiber	Anthocyanin	pH‐sensitive	—	TS: 23.95–14.68 MPa	—	No significant change in thermal stability	Pink at pH 2–4, light pink at pH 5, light dark green at pH 10	DPPH scavenging rate 5.3%–49.06%	Pink nanofiber changed to powder blue color as fresh fish spoiled, producing reddish brown color on its surface	Duan et al. (2021)
					EB: 7.45%–24.55%				Slight antibacterial activity		

### 9.4 Pullulan in Intelligent Packaging

Intelligent packaging involves the incorporation of a substance into the packaging that engages with the surroundings and actively monitors the quality of the packaged food (Du et al. 2025). It possesses the ability to identify and indicate the decay in quality or rise of any potential concerns about the food packed. In a broader sense, intelligent materials can be classified into two categories: internal indicators and external indicators. For instance, the gas sensors, food freshness indicators, and microbial indicators are typically attached inside the food package to ensure direct contact with the compounds emitted by the food (Cheng et al. 2022). Conversely, the barcodes and time‐temperature indicators are structurally positioned on the exterior of the food package to document alterations in the external environment throughout the storage period and convey the current status to consumers (Azeredo and Correa 2021).

The anthocyanin compound exhibits varying hues across different pH levels, with pink, light pink, lavender, light blue, dark green, light dark green, and yellow‐green at pH 2–4, 5, 6, 7, 8, 9, 10, and 11, respectively. With the help of these features, Duan et al. (2021) managed to detect spoilage of *Plectorhynchus cinctus* fish by packing it into anthocyanin‐loaded pullulan and chitosan nanofiber. The primary reason for seafood spoilage is decomposition of proteins, facilitated by enzymatic and microbial activity, resulting in the production of volatile nitrogen compounds. The presence of nitrogen compounds has the potential to elevate the pH levels within the food packaging environment. The researchers observed that color transition of anthocyanin‐added pullulan‐based nanofiber was vivid to different pH values, as evidenced by darkness of green color in the fiber beyond pH 8. These results further helped to monitor the spoilage of *P. cinctus* fish as the pink color of the nanofiber changed to powder blue after 72 h storage at refrigerated conditions. Consequently, the pH of the fish exceeded 7, and the tight texture of the fish turned soft, indicating the spoilage. Unfortunately, the authors did not investigate the amount of nitrogen released from fish, which is a crucial indicator for fish spoilage. In another study, nisin and red cabbage anthocyanin exhibited dynamic visual monitoring of the freshness through color response in packed fish (Wu et al. 2022). Similar to previous findings, they observed that red cabbage anthocyanin added to pullulan‐based film exhibited a halochromic action as the total color difference of film increased by 11% as pH exceeded 7, but with increase in concentration of anthocyanin in film, total color difference decreased at the same level, as excess concentration may obscure the color change to different pH values. The researchers were effectively alerted to the potential spoilage of sea bass as blue color of intelligent film transitioned to blue‐green and yellow‐green after 48 and 96 h, respectively. Similar to previous research, they also concluded the spoilage of sea bass based on the film's color change throughout storage without performing tests on TVB‐N to quantitatively evaluate the spoilage. Similarly, Bian et al. (2024) also used anthocyanin‐rich *Broussonetia papyrifera* fruit extract in pullulan and gellan gum film to monitor the freshness of *Pelteobagrus fulvidraco* fish at 4°C. In contrast, they reported that the TVB‐N content in fish increased by 417%, reaching 31 mg/100 g after 72 h of storage at 4°C. According to the Chinese standard, 30 mg/100 g TVB‐N content is the freshness threshold for fish. Consequently, the red color of film changed to yellowish‐grey color after 24 h of storage, as the fish spoiled.

## 10 Regulatory and Safety Considerations for Pullulan‐Based Packaging

Pullulan had been evaluated by regulatory authorities for its safety when used as a food additive and for its packaging applications. In the United States, the FDA classifies pullulan as a permitted food additive under 21 CFR 172.240 regulation and recognizes it as GRAS due to its nontoxic and noncarcinogenic nature (21 CFR Part 172, 2025). On the other hand, within the European Union, the European Food Safety Authority (EFSA) authorizes pullulan as a food additive, E 1204, and reports no safety concerns at approved levels, except for mild gastrointestinal effects, such as bloating or cramping, at intake above 10 g/day (EFSA Panel on Food Additives and Flavourings (FAF) et al. 2025). The International Organization for Standardization (ISO) reports globally harmonized frameworks to ensure the quality and safety of food‐contact materials (Gupta et al. 2024). ISO standard—ISO 22000:2018—highlights food safety management systems, ensuring biopolymers meet hazard analysis and critical control point principles across the food supply chain (Dabija et al. 2024). Additionally, ISO 18604:2013 focuses on material recycling and safety for sustainable use of biopolymers in packaging (Maria Mosconi et al. 2024). ISO test standards for overall and specific migration provide analytical techniques to evaluate potential contaminant transfer under realistic storage conditions (Störmer et al. 2024).

Although native pullulan films show minimal additive migration (Ghosh et al. 2022), incorporating plasticizers, crosslinkers, nanofillers, or bioactive compounds can modify their migration profiles (Milenkovi and Nikoli 2021). Therefore, safety evaluation under realistic time‐temperature and food‐simulant conditions for each film type remains essential. Moreover, chemical modifications such as esterification, oxidation, amidification, or blending with other polymers warrant careful monitoring of potential residual reagents, degradation products, and any toxicity endpoints (Varadhan and Jayaraman 2024; Selvakumar and Lonchin 2020). Furthermore, when pullulan‐based packaging incorporates plant‐derived actives, interactions with food should not affect its nutritional or sensory attributes. Overall, pullulan's regulatory acceptance by both FDA and EFSA underscores its suitability for edible and food‐contact packaging, though continuous monitoring of emerging modifications and nanocomposite formulations is required to maintain compliance.

## 11 Key Challenges

Despite significant laboratory‐scale success, the commercial transition of pullulan‐based packaging is challenged by hurdles associated with industrial‐scale production, downstream processing, and diversity of environmental conditions to which pullulan‐based films will be exposed. The high production cost is primarily due to the limited yield and metabolic instability of *A. pullulans*, where carbon flux may divert toward by‐products such as melanin and polymalic acid. (Aquinas et al. 2024). In addition, the organism frequently undergoes morphological shifts and metabolic divergence owing to its dimorphic nature and heterogeneous population structure, which diverts carbon flux away from pullulan biosynthesis (Cruz‐Santos et al. 2023). Furthermore, prolonged subculturing and continuous fermentation can exacerbate this instability through mutations or loss of biosynthetic capacity, resulting in a gradual decline in productivity over successive cultivation cycles. Additionally, high broth viscosity and limited oxygen transfer reduce fermentation efficiency, making large‐scale production difficult. Processing inefficiency represents another major bottleneck. Pullulan's low thermal stability prevents its suitability for conventional extrusion and melt‐processing methods involved in film formation, whereas its hydrophilic nature leads to water absorption, swelling, and mechanical weakening under humid conditions. These drawbacks necessitate blending or chemical modification to improve thermal and moisture resistance (as discussed in the preceding sections). From a regulatory and sustainability perspective, standardized safety guidelines for modified or nanocomposite pullulan films are still lacking, and data on their toxicity, migration, and end‐of‐life management remain largely unexplored. Although pullulan is biodegradable under controlled conditions, its industrial compostability, recycling compatibility, and life cycle environmental performance require systematic evaluation. Similarly, the feasibility of incorporating pullulan films into existing mechanical or chemical recycling streams is unclear, as their solubility, hydrophilicity, and potential interactions with other polymers may hinder degradation or recycling efficiency, raising questions about their end‐of‐life fate and overall environmental benefits. Together, these challenges create a significant gap between academic research and commercial translation.

## 12 Future Research Directions

Future research should prioritize the development of cost‐effective and scalable production systems to overcome the economic limitations of pullulan. One promising avenue lies in the valorization of agro‐industrial byproducts as alternative feedstocks, which could reduce dependence on refined sugars and enhance process sustainability. In parallel, advances in fermenting strain improvement, through classical mutagenesis or modern genetic engineering, are needed to generate microbial strains with higher pullulan yields, improved metabolic stability, and greater tolerance to industrial stress conditions. Such integrated strategies will not only lower production costs but also support the transition from laboratory‐scale studies to industrial‐scale implementation.

Future studies should explore advanced material engineering approaches such as surface modification (e.g., esterification, etherification, oxidation, and amidification) and strategic blending with compatible biopolymers to mitigate the inherent moisture sensitivity and lack of functional properties of pullulan. These interventions can potentially reduce water absorption and swelling while enhancing water barrier properties and the functional characteristics of pullulan‐based films under real‐world packaging conditions. Advanced fabrication techniques present promising opportunities to expand the applicability of pullulan‐based packaging. For instance, 3D printing enables high structural precision and customizable geometries, allowing the spatial incorporation of active substances or sensing entities for multifunctional packaging solutions (Tracey et al. 2022). Similarly, extrusion‐based processing holds the greatest potential for large‐scale manufacturing, given its industrial compatibility and high throughput, though challenges related to the thermal instability of pullulan must be resolved through blending, plasticization, or chemical modification. Despite solution casting, electrospinning of pullulan offers a versatile platform for fabricating nanofibrous mats with high surface area, tunable porosity, and superior barrier properties, making them particularly suitable for incorporating bioactive compounds, reinforcing agents, or natural indicators to enhance both the functional and intelligent attributes of packaging.

On the other hand, the integration of artificial intelligence (AI) with pullulan‐based packaging offers a transformative pathway toward truly intelligent food packaging systems (Yakoubi 2025). The integration of natural halochromic indicators (e.g., anthocyanin, betalain, and curcumin) into pullulan films with digital technologies, such as biosensors, Radio Frequency Identification (RFID) systems, and cloud‐based platforms, enables the application of AI for real‐time food quality monitoring, predictive shelf‐life assessment, and supply chain optimization. Long‐term migration studies and comprehensive toxicological assessments must be prioritized, especially for pullulan composites containing nanoparticles or chemical crosslinkers, to ensure regulatory acceptance. Environmental sustainability assessments, including life cycle analysis, biodegradation studies of pullulan‐based films under industrial composting conditions, and evaluation of recycling pathways, are critical to establish pullulan as a truly green alternative.

## 13 Conclusions

Over the past decade, extensive research has highlighted pullulan as a highly promising biopolymer for food packaging and edible coatings. Its unique characteristics, including edibility, transparency, odorlessness, excellent OB properties, heat sealability, and flexibility, make it well‐suited for food‐related applications. Despite these excellent properties, the inherently brittle nature and limited EB of pullulan‐based films are restrictive to standalone use in commercial packaging. The incorporation of high MW plasticizers (e.g., sorbitol and polyethylene glycol) disrupts intermolecular hydrogen bonding within pullulan chains, enhancing their mobility and flexibility. In addition, blending with other biopolymers (e.g., polyvinyl alcohol and polylactic acid), chemical crosslinkers (e.g., citric acid), and nanofillers (e.g., cellulose nanocrystals) strengthens native pullulan matrix by developing intermolecular networks. Moreover, advanced fabrication techniques, such as electrospinning and multilayer assembly, have potential to enhance the mechanical properties of pullulan‐based films. On the other hand, the high moisture sensitivity of pullulan can be addressed by esterifying pullulan for its broad application in food packaging. In addition, amidification of the pullulan surface can impart tailored functional properties to the native pullulan films. However, although chemical modifications and composite formulations offer tailor‐made properties, concerns about the safety and potential toxicity of transformed pullulan in food contact applications warrant further investigations. Beyond passive packaging, pullulan has gained traction in active packaging applications through the incorporation of antimicrobial agents, bioactive compounds, and engineered nanomaterials. These enhancements improve the functional performance of the packaging and contribute to shelf life extension. Furthermore, encapsulation techniques, including MOF, Pickering emulsions, nanoemulsions, and nanoliposomes, have been effectively employed to stabilize and control the release of active substances within pullulan matrices.

## Author Contributions

**Bibek Bahadur Shrestha**: data curation, formal analysis, investigation, methodology, software, visualization, writing–review and editing, writing–original draft. **Jayeeta Mitra**: conceptualization, investigation, funding acquisition, project administration, software, visualization, supervision, writing–review and editing, writing–original draft. **Saji George**: conceptualization, investigation, project administration, resources, supervision, visualization, software, writing–original draft, writing–review and editing, funding acquisition.

## Conflicts of Interest

The authors declare no conflicts of interest.
